# Mouse TRIP13/PCH2 Is Required for Recombination and Normal Higher-Order Chromosome Structure during Meiosis

**DOI:** 10.1371/journal.pgen.1001062

**Published:** 2010-08-12

**Authors:** Ignasi Roig, James A. Dowdle, Attila Toth, Dirk G. de Rooij, Maria Jasin, Scott Keeney

**Affiliations:** 1 Molecular Biology Program, Memorial Sloan-Kettering Cancer Center, New York, New York, United States of America; 2 Gerstner Sloan-Kettering Graduate School of Biomedical Sciences, New York, New York, United States of America; 3 Institute of Physiological Chemistry, Technische Universität Dresden, Dresden, Germany; 4 Center for Reproductive Medicine, Amsterdam Medical Center, University of Amsterdam, Amsterdam, Netherlands; 5 Developmental Biology Program, Memorial Sloan-Kettering Cancer Center, New York, New York, United States of America; 6 Howard Hughes Medical Institute, Memorial Sloan-Kettering Cancer Center, New York, New York, United States of America; National Cancer Institute, United States of America

## Abstract

Accurate chromosome segregation during meiosis requires that homologous chromosomes pair and become physically connected so that they can orient properly on the meiosis I spindle. These connections are formed by homologous recombination closely integrated with the development of meiosis-specific, higher-order chromosome structures. The yeast Pch2 protein has emerged as an important factor with roles in both recombination and chromosome structure formation, but recent analysis suggested that TRIP13, the mouse Pch2 ortholog, is not required for the same processes. Using distinct *Trip13* alleles with moderate and severe impairment of TRIP13 function, we report here that TRIP13 is required for proper synaptonemal complex formation, such that autosomal bivalents in *Trip13*-deficient meiocytes frequently displayed pericentric synaptic forks and other defects. In males, TRIP13 is required for efficient synapsis of the sex chromosomes and for sex body formation. Furthermore, the numbers of crossovers and chiasmata are reduced in the absence of TRIP13, and their distribution along the chromosomes is altered, suggesting a role for TRIP13 in aspects of crossover formation and/or control. Recombination defects are evident very early in meiotic prophase, soon after DSB formation. These findings provide evidence for evolutionarily conserved functions for TRIP13/Pch2 in both recombination and formation of higher order chromosome structures, and they support the hypothesis that TRIP13/Pch2 participates in coordinating these key aspects of meiotic chromosome behavior.

## Author Summary

Meiosis is the specialized cell division that gives rise to reproductive cells such as sperm and eggs. During meiosis in most organisms, genetic information is exchanged between homologous maternal and paternal chromosomes through the process of homologous recombination. This recombination forms connections between homologous chromosomes that allow them to segregate accurately when the meiotic cell divides. Recombination defects can result in reproductive cells with abnormal chromosome numbers, which are a major cause of developmental disorders and spontaneous abortions in humans. Meiotic recombination is tightly controlled such that each pair of chromosomes undergoes at least one crossover recombination event despite a low average number of crossovers per chromosome. Recombination is coordinated with the development of specialized, meiosis-specific chromosome structures that stabilize pairing interactions between homologous maternal and paternal chromosomes. We show here that the mouse TRIP13 protein is required for normal execution of many aspects of meiotic recombination and chromosome structure development that it was not previously known to influence. Intriguingly, many of these new roles appear to parallel known functions of a homologous protein from budding yeast, called Pch2. These findings thus indicate that TRIP13/Pch2 functions are more widely conserved throughout evolution than thought before.

## Introduction

Meiosis generates haploid gametes from a diploid progenitor in order for proper ploidy to be restored after fertilization. Meiocytes accomplish haploidization by performing two rounds of chromosome segregation after a single round of replication. During prophase of the first meiotic division in most organisms, DNA double-strand breaks (DSBs) are produced by the Spo11 protein [1] and repair of these breaks promotes recombination, pairing, and synapsis of homologous chromosomes [2]. Meiotic recombination can lead to a crossover (CO), involving reciprocal exchange of chromosome arms flanking the break, or to a non-crossover (NCO). COs, in conjunction with sister chromatid cohesion, provide physical connections between homologous chromosomes that ensure their faithful segregation in the first meiotic division.

As recombination progresses, chromosomes form higher order structures, most prominently the synaptonemal complex (SC), which comprises two lateral elements (one for each homologous chromosome) and transverse filaments linking them together [3], [4]. In many species, mutations affecting chromosome structure components perturb meiotic recombination, and, conversely, mutants defective for recombination proteins have chromosome structure defects (reviewed in [5], [6]). These and other observations demonstrate the interrelatedness of recombination and higher order chromosome structures [6].

In budding yeast, Pch2 (pachytene checkpoint 2) is a meiosis-specific AAA+ ATPase family member that is required for checkpoint arrest in response to certain meiotic defects, including those caused by absence of the SC transverse filament protein Zip1 [7], [8]. This checkpoint role depends specifically on a sub-population of Pch2 protein that localizes within the nucleolus [7], [9]. Checkpoint-related roles have also been observed in *C. elegans*, where *pch-2* is required for apoptosis of oocytes in mutants deficient for SC components [10] and in *D. melanogaster*, where *pch2* is required for a delay in oocyte selection that occurs in mutants defective for certain crossover-promoting factors [11].

More recently, a chromosomally localized fraction of yeast Pch2 has been shown to play important roles in normal (unperturbed) meiosis. First, Pch2 is required for timely and efficient recombination: DSBs persist longer in *pch2* mutants than in wild type [12]; *pch2* mutants show a slight delay in meiotic divisions that is dependent on Rad17, a checkpoint factor that responds to unrepaired DSBs [13]; and *pch2* mutants are delayed for formation of both COs and NCOs [9], [13]. Second, Pch2 is important for CO control: *pch2* mutants are defective in maintaining normal separation of adjacent COs (“CO interference”), maintaining wild-type numbers of COs when meiotic DSBs are reduced by hypomorphic *spo11* mutations (“CO homeostasis”), and ensuring formation of at least one CO per chromosome pair (the “obligate CO”) [14], [15]. Third, Pch2 is required for proper formation and/or maintenance of SC or other higher order chromosome structures: *pch2* mutants show abnormal chromosomal localization of the SC central element protein Zip1 and the axis-associated protein Hop1 [9], [14]. Because Pch2 is needed for both recombination and chromosome structure formation, Pch2 has been hypothesized to coordinate these two features of meiotic chromosome dynamics [9], [14], [15].

In mouse, a hypomorphic mutation of the *PCH2* ortholog, *Trip13* (thyroid hormone receptor interacting protein), supports apparently normal apoptosis of recombination- or synapsis-defective mutants, suggesting that checkpoint functions of TRIP13 are not conserved in mammals [16]. However, TRIP13 is essential for completion of otherwise wild-type meiosis in both male and female mice. Interestingly, mutant spermatocytes were defective for completing meiotic DSB repair but were competent to complete homologous synapsis and appeared to form normal numbers of COs. These observations led to the suggestion that, unlike Pch2 in yeast, TRIP13 is involved specifically in a recombination pathway(s) that leads to NCOs, but is dispensable for COs [16]. These findings thus suggested that Pch2/TRIP13 plays different roles in mouse than in other organisms.

Here we present characterization of a more severe *Trip13* mutant allele along with more detailed analysis of the previously described hypomorph. These studies reveal for the first time that TRIP13 is needed for efficient completion of homologous synapsis. Moreover, we provide evidence that TRIP13 promotes early steps of the DSB repair process upstream of the assembly of RAD51 complexes, and is required for normal number and distribution of COs, thus affecting both CO and NCO pathways. The TRIP13 functions revealed in this study are reminiscent of many of the functions observed for the chromosome-bound Pch2 protein in budding yeast, implying evolutionarily conserved roles.

## Results

### *Trip13* mutant mice

**Figure 1:**
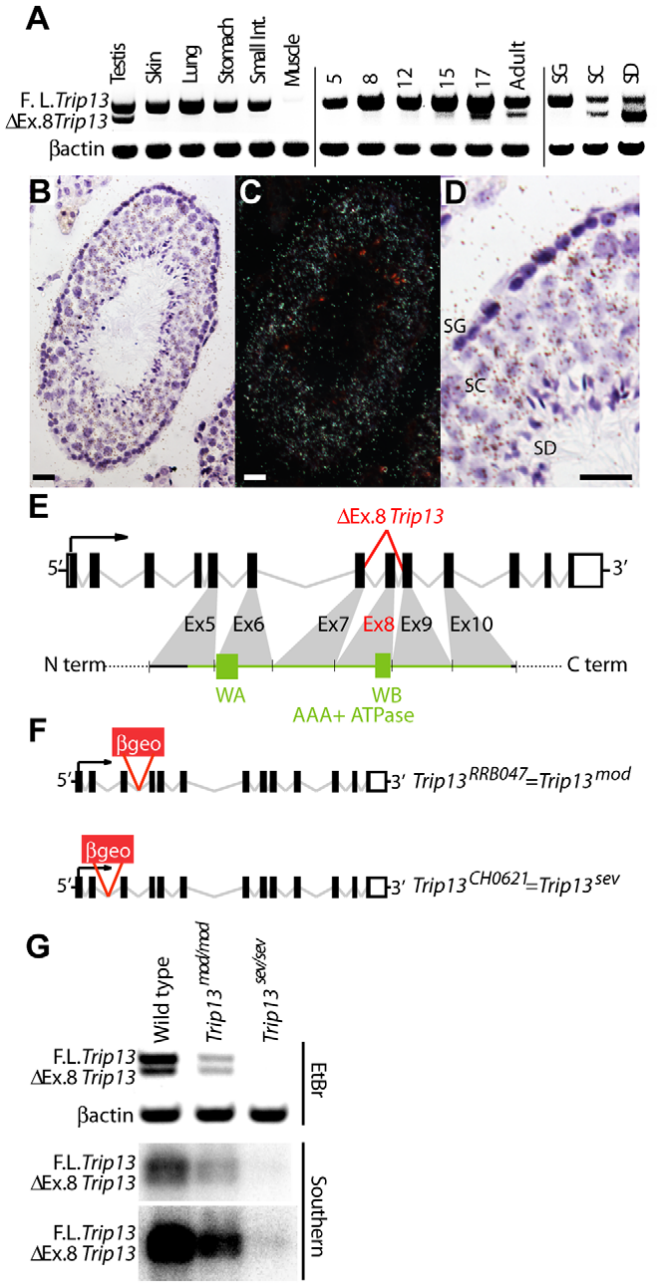
*Trip13* expression in mouse. (A) Left panel: *Trip13* expression in different mouse tissues detected by RT-PCR from a commercial set of cDNAs (Origene). β actin expression served as a control. In addition to full-length transcript, a smaller transcript was identified lacking exon 8 (Δ*Ex.8 Trip13*) that was abundant in adult testis, but was at trace levels or absent in other tissues. The small size difference (87 bp) may explain why this isoform was not previously detected. Middle and right panels: *Trip13* expression detected by RT-PCR on samples from whole testes of juveniles of the indicated ages (in dpp) and flow-sorted populations from adult testes, enriched for the indicated cell types (spermatogonia (SG), spermatocytes (SC), and spermatids (SD)). RT-PCR analysis during the first, semi-synchronous meiotic wave in juvenile testes detected the longer *Trip13* transcript in all samples analyzed, including pre-meiotic samples (5 dpp). In contrast, Δ*Ex.8 Trip13* was detected only in samples containing cells that had already entered meiosis (12 dpp onwards; meiotic prophase starts ∼8 dpp). RT-PCR on flow-sorted, highly enriched cell populations from adult testis detected full-length *Trip13* transcript in all cell types analyzed, but Δ*Ex.8 Trip13* was seen only in meiotic (spermatocytes) and post-meiotic cell types (spermatids). *Trip13* is thus expressed throughout spermatogenesis, but with developmentally regulated exclusion of exon 8 via alternative splicing. (B–D) RNA *in situ* hybridization to a testis section. The *Trip13* hybridization signal appears as dark grains in the bright field image of a seminiferous tubule (B) and white grains in the corresponding dark field image (C). A zoomed image of the same tubule (D) shows *Trip13* hybridization signal overlaying spermatogonia (SG), spermatocytes (SC) and spermatids (SD). Bars  = 20 µm. (E) Schematic showing *Trip13* splicing variants as well as contribution from exons 5–10 to the AAA+ ATPase domain. Walker A and B motifs (WA and WB) are depicted as green boxes. The Δ*Ex.8 Trip13* transcript maintains the open reading frame, but lacks sequences for the Walker B ATPase motif. (F) Schematic of the *Trip13* alleles used in this study. Red boxes indicate gene trap insertion locations. (G) RT-PCR analysis of *Trip13* expression in whole-testis samples from wild-type and mutant animals. PCR products were detected by ethidium bromide staining (EtBr) or Southern blotting (two exposures of the Southern blot are shown). Only the full-length *Trip13* transcript is detected in *Trip13^sev/sev^* testes, which may be a consequence of later stage germ cells being absent in this mutant (see Figure 1A).

*Trip13* is widely expressed in adult tissues, including testis [16] (Figure 1A), where it is expressed in spermatogonia, spermatocytes and spermatids (Figure 1A–1D). A splice variant lacking exon 8 (which includes the Walker B ATPase motif) was detected specifically in spermatocytes and spermatids (Figure 1A, 1E, 1G), although the functional significance of this form of the *Trip13* mRNA is not yet clear.

We created *Trip13* mutant mice using ES cell lines containing a gene trap in either intron 2 (clone CH0621) or intron 3 (clone RRB047) (Figure 1F). These alleles yielded different phenotypes. As described by Li and Schimenti [16], *Trip13^RRB047^* is a hypomorphic mutation that significantly reduces *Trip13* expression, such that transcripts are detected by RT-PCR at reduced levels in *Trip13^RRB047/RRB047^* mice (Figure 1G). *Trip13* expression was even more substantially reduced in testes from *Trip13^CH0621/CH0621^* mice (Figure 1G). *Trip13^CH0621^* is thus significantly tighter than *Trip13^RRB047^*. For clarity, we refer below to *Trip13^RRB047^* as *Trip13^mod^* (for “moderate”), and to *Trip13^CH0621^* as *Trip13^sev^* (for “severe”). Unless otherwise noted, control mice were littermates homozygous for the wild-type allele, and all analyses were conducted on at least two mutant/control pairs to ensure reproducibility.

Both *Trip13* alleles segregated in a sub-Mendelian ratio: only 11.5% of pups from *Trip13^+/mod^* mice were homozygous mutants (32.5% wild type and 56.0% heterozygotes, N = 416), in agreement with prior results [16]. Similarly, only 10.9% *Trip13^sev/sev^* homozygotes were obtained from *Trip13^+/sev^* crosses (30.7% wild type and 58.4% heterozygotes, N = 202). Segregation of the mutant alleles is not significantly different (p = 0.956, G test). The sub-Mendelian inheritance suggests that TRIP13 has another function apart from its role in meiosis. *Trip13^mod/mod^* animals in certain strain backgrounds were found to have reduced body size and abnormal tails [16], although we observed no obvious somatic phenotypes for either allele in our colony (data not shown). This difference between the two studies is likely attributable to effects of strain background on expressivity and penetrance of somatic phenotypes.

### TRIP13 is needed for completion of meiosis

**Figure 2:**
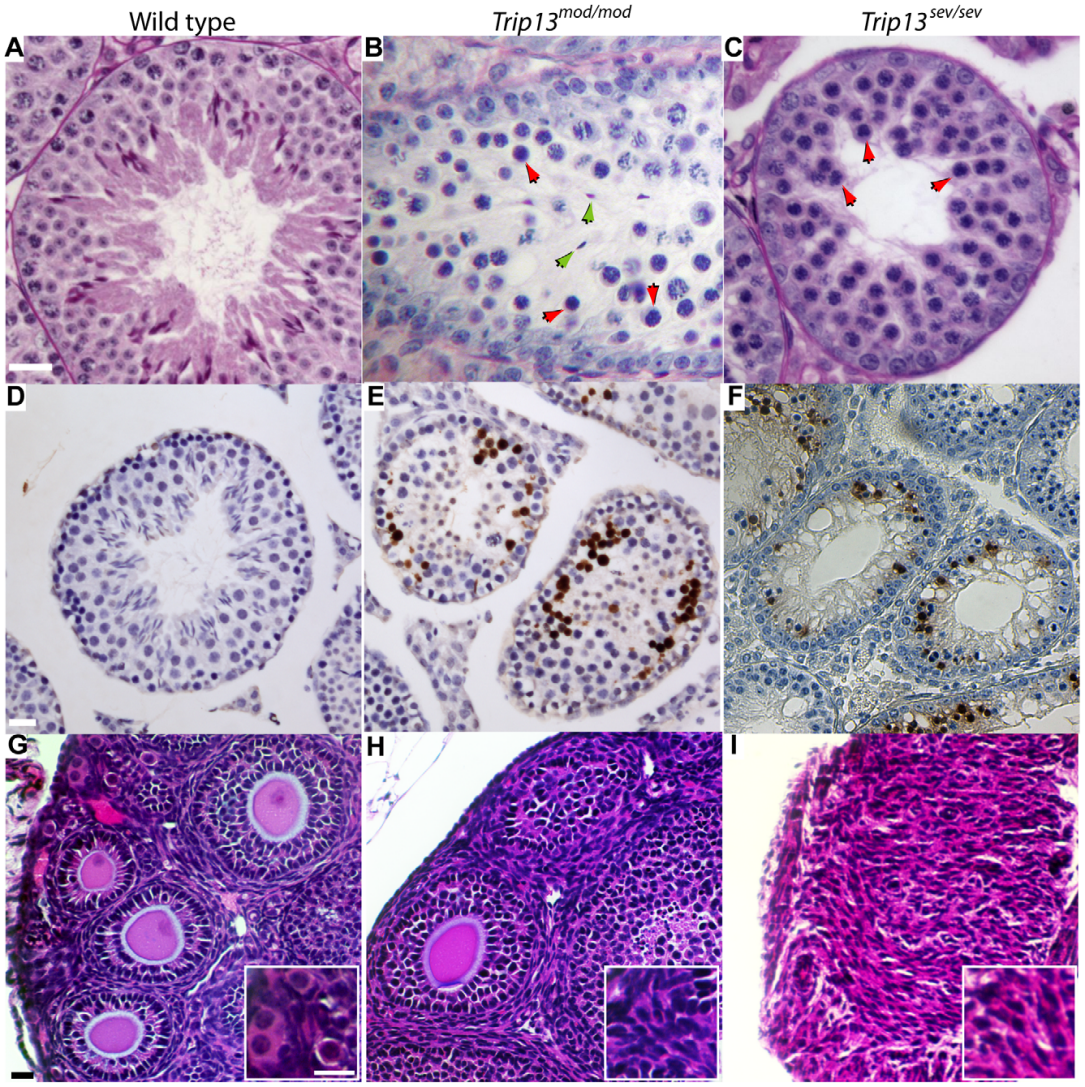
TRIP13 is needed for completion of meiosis in males and females. (A–C) Representative epithelial stage IV tubules from periodic acid Schiff (PAS)-stained testis sections from mice of the indicated genotypes. Red arrowheads indicate pachytene spermatocytes with an apoptotic morphology (condensed, darkly staining nuclei). Green arrowheads point to examples of postmeiotic cells (elongating spermatids) seen infrequently in *Trip13^mod/mod^* mutants but not observed in *Trip13^sev/sev^* mutants. (D–F) TUNEL-stained testis sections from mice of the indicated genotypes. In wild type (D), few TUNEL-positive cells (stained brown) were seen, most often spermatogonia. In both *Trip13* mutants (E, F), numerous TUNEL-positive spermatocytes were found. (G–I) PAS-stained ovary sections from 21-day-old mice. In wild type, several follicles at different stages of development can be observed, including primordial follicles (inset in Panel G). In contrast, *Trip13^mod/mod^* ovaries had no primordial follicles (inset in Panel H) and fewer growing follicles (19.3±4.7 per section vs. 75.8±16.2 in wild type). *Trip13^sev/sev^* ovaries completely lacked follicles (panel I). Each bar applies to all images in the row and represents 20 µm.

As previously reported [16], we found that TRIP13 is needed for the completion of meiosis, since homozygous mutant animals were sterile because of severe blocks to spermatogenesis or oogenesis. For both alleles, mutant males had significantly smaller testes than wild-type littermates (0.59±0.16% of total body mass in wild type (avg. ± sd, N = 8); 0.28±0.22% in *Trip13^mod/mod^* (N = 10); and 0.14±0.01% for *Trip13^sev/sev^* (N = 4); p ≤ 0.05 for either mutant vs. wild type, t test; difference between mutants was not significant (p = 0.225)). Testes contain seminiferous tubules, within which germ cells undergo spermatogenesis. Tubule cross sections can be classified in stages, from I–XII, based on the array of germ cell developmental steps present [17]. Histological analysis revealed that testes from both mutants had less populated tubules compared to wild type (Figure 2A–2C). In *Trip13^mod/mod^* mice, spermatogenesis was mostly arrested at spermatocyte stages of epithelial stage IV, corresponding to pachynema (red arrowheads, Figure 2B). However, spermatocytes occasionally escaped this arrest as judged by presence of some post-meiotic cells (green arrowheads, Figure 2B). However, we did not observe any late-stage spermatids, implying that cells that escape pachytene arrest could not complete spermiogenesis. TUNEL staining revealed that most of the cells arrested at epithelial stage IV undergo apoptosis (Figure 2E). *Trip13^sev/sev^* males also arrested spermatogenesis at epithelial stage IV (Figure 2C and 2F), but no post-meiotic cells were observed, indicating that pachytene arrest is tighter.

Oogenesis was also severely affected by both mutations. Female germ cells complete early stages of meiotic prophase I during fetal development and undergo cell cycle arrest at around birth. Soon after birth, each surviving oocyte becomes surrounded by somatic (granulosa) cells, forming a primordial follicle that remains arrested until recruited for further development during the reproductive life of the animal [18]. At this time, some follicles undergo an initial synchronous wave of development, in which the oocyte increases in size and the somatic cells change shape and proliferate (Figure 2G). Ovaries from 21-day-old *Trip13^mod/mod^* animals had no detectable primordial follicles and had four-fold fewer developing follicles than wild type (Figure 2H). By two months of age, no follicles were detected in this mutant (data not shown). These results indicate that a large majority of *Trip13^mod/mod^* oocytes are unable to complete folliculogenesis; the small number of escapers that generate follicles are immediately recruited to develop, causing rapid depletion of the oocyte pool. Ovaries from *Trip13^sev/sev^* females showed an even greater defect, as they were devoid of follicles at 21 dpp (Figure 2I). A block to folliculogenesis is typical of mutants unable to repair meiotic DSBs [19], suggesting that TRIP13 is required for completion of meiotic recombination (see below).

### *Trip13* mutants display unusual defects in homologous synapsis of autosomes

**Figure 3:**
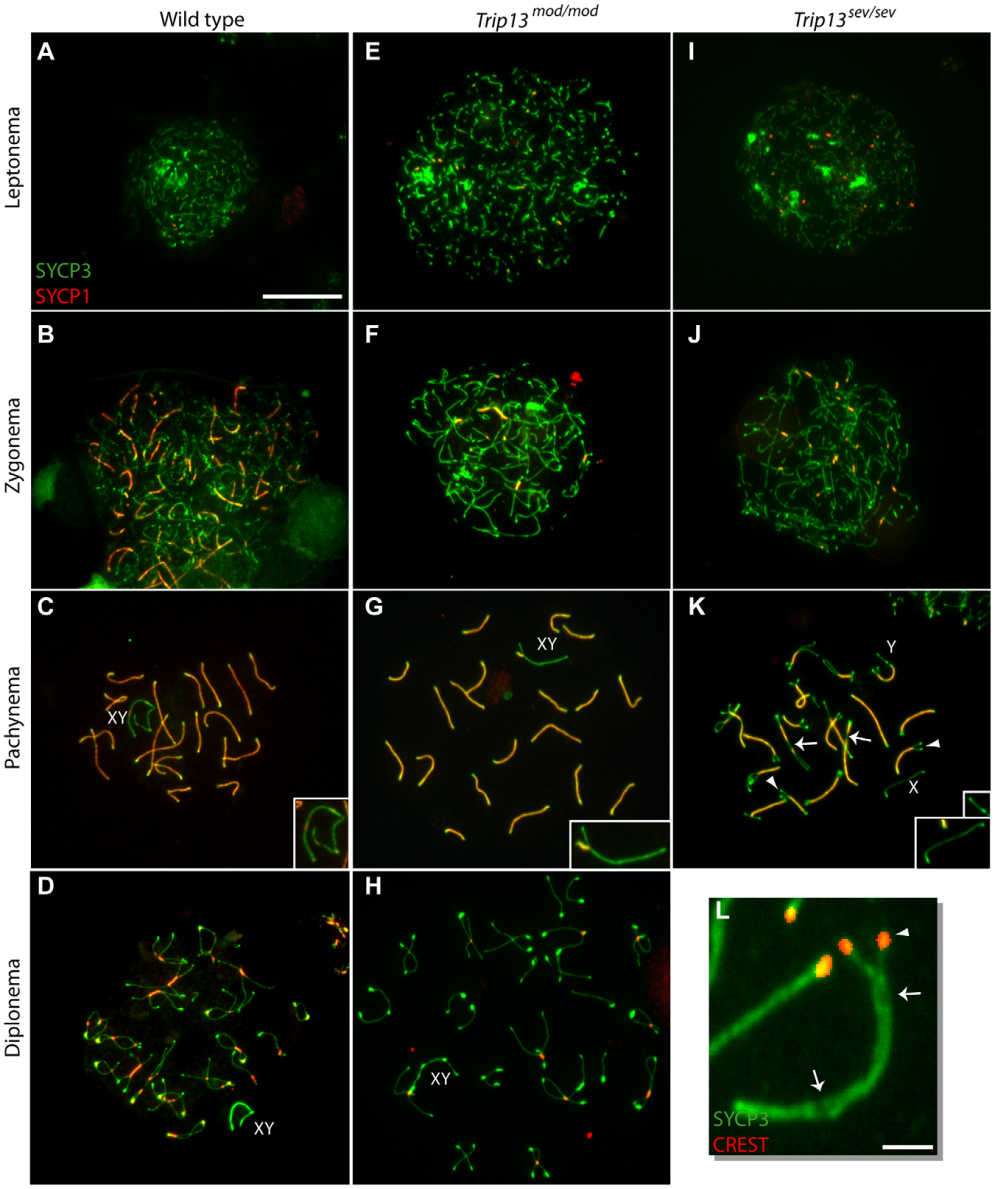
TRIP13 is required to complete synapsis in spermatocytes. (A–K) Progression of synapsis followed by staining spermatocyte chromosome spreads for SYCP3 (green) and SYCP1 (red). *Trip13^mod/mod^* spermatocytes (E–H) show progression of synapsis (leptonema through pachynema) and subsequent desynapsis (diplonema) that is largely comparable to wild type (A–D). In contrast, *Trip13^sev/sev^* spermatocytes form axial elements (I) and initiate synapsis (J) but never achieve complete synapsis of all homologs (K). The cell in (K) is a representative example of the most advanced stage observed in the *Trip13^sev/sev^* mutant and has characteristics of both zygonema and pachynema (see text). Synapsed X and Y chromosomes are indicated in (C–D) and (G–H); they are unsynapsed in (K). Bar on (A) applies to (A–K) and represents 20 µm. (L) Zoomed image of a selected SC with pericentric fork (i.e., an unsynapsed centromeric end, as detected by CREST staining) and interstitial unsynapsis. Bar = 1.5 µm. Arrowheads in (K, L) point to examples of bivalents whose ends have failed to complete synapsis; arrows point to examples of interstitial asynapsis.

During meiotic prophase, homologous chromosome pairing is stabilized by the SC. Synapsis can be monitored by following deposition of proteins of the lateral elements (e.g., SYCP3) and the central element (e.g., SYCP1) by immunofluorescence on spread chromosomes. In wild-type meiosis, stretches of SYCP3 first appear at leptonema (Figure 3A). Later, at zygonema, homologs begin to synapse, marked by the presence of SYCP1 (Figure 3B). At pachynema, homologous autosomes are completely synapsed along their lengths such that SYCP3 and SYCP1 completely co-localize (Figure 3C). At diplonema, homolog axes separate but remain joined at sites where COs have occurred, called chiasmata (Figure 3D).

In *Trip13^mod/mod^* spermatocytes, progression of autosomal synapsis appeared similar to wild type [16] (Figure 3E–3G), and at diplonema, homologous axes separated from one another normally, with most remaining joined at one or a few sites, consistent with chiasmata (Figure 3H). However, a more detailed analysis revealed that SC formation was not fully normal. Specifically, total autosomal SC length at pachynema was on average 11% shorter than in wild type (152.2±8.5 µm in *Trip13^mod/mod^* (N = 20 cells) vs. 171.6±15.7 µm in wild type (N = 32 cells); p = 0.0001, t test). To test if shorter SCs affected only chromosomes of a particular size range, we measured the length of all bivalents, ranked them by size, and then divided them into five groups of similarly sized chromosomes (Table 1). For each chromosome group, the average length of an SC in the mutant was significantly smaller than in wild type. Thus, all autosomal SCs were reduced in length to a similar extent.

**Table 1:** Autosomal SCs are shorter on average in *Trip13^mod/mod^* spermatocytes.

	Wild type			*Trip13^mod/mod^*		
Chromosome size ranks	SC length (µm)^a^	% of total SC^b^	N^c^	SC length (µm)a,d	% of total SC^b^	N^c^
1–2	12.8±1.7	7.5	22	11.0±1.0	7.4	34
3–5	11.1±1.2	6.5	33	9.5±0.7	6.4	51
6–11	9.5±1.1	5.5	66	8.1±0.7	5.5	102
12–16	7.7±0.9	4.5	55	6.8±0.6	4.6	85
17–19	5.8±1.3	3.4	33	5.2±1.0	3.5	51

Pachytene spermatocyte spreads were immunostained for SYCP3 and lengths of autosomal SCs were measured from wild-type and *Trip13^mod/mod^* spermatocytes. The bivalents in each spread were rank-ordered by length from 1 (largest) to 19 (smallest), then divided into groups of similarly sized chromosomes. Note that each of the chromosomes in each size class makes up essentially the same percentage of total SC length in both genotypes, indicating that the decrease in average total SC length in *Trip13^mod/mod^* spermatocytes is uniform across all autosomes.

a Mean ± standard deviation for each chromosome in the indicated size rank.

b The mean SC length per chromosome divided by the average total SC length per cell, multiplied by 100.

c Total number of chromosomes analyzed.

d These values are statistically significantly different from those for wild type (p≤0.05, t test).

**Figure 4:**
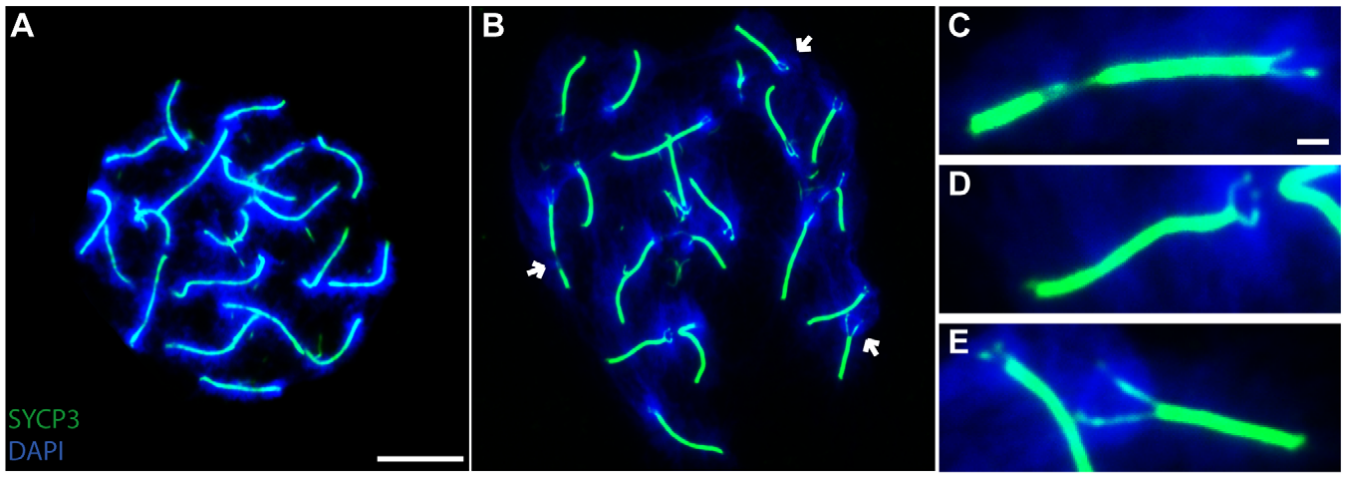
Synapsis defects in TRIP13-deficient oocytes. Spread chromosomes from a wild-type pachytene oocyte (A) or a *Trip13^sev/sev^* pachytene-like oocyte (B–E) stained for SYCP3 (green) and DNA (DAPI, blue). Arrows indicate examples of unsynapsed regions, zoomed images of which are shown in (C–E). Bar on (A) applies to (A, B) and represents 10 µm. Bar on (C) applies to (C–E) and represents 1 µm.

These findings indicate that wild-type levels of TRIP13 are needed for formation of structurally normal SC, but the defects in *Trip13^mod/mod^* mutants were relatively subtle. If these modest defects reflect a bona fide requirement for TRIP13, then more substantial defects should be encountered in *Trip13^sev/sev^* cells. This was in fact the case. Synapsis initiation appeared normal in *Trip13^sev/sev^* spermatocytes, as judged by staining patterns for SYCP3 and SYCP1 in leptonema and zygonema (Figure 3I–3J), but proper pachynema with fully synapsed autosomal bivalents was never observed. Instead, the most advanced cells showed a late zygotene-like morphology—given that full-length axes had developed but remained incompletely synapsed—but with characteristics of pachytene cells such as acquisition of the characteristic knob-like accumulation of SYCP3 at telomeres (Figure 3K and Table 2). Because there does not appear to be a stage when synapsis is complete, it is likely that the unsynapsed regions are asynaptic (i.e., where SC did not form at all) rather than desynaptic (i.e., where SC had formed but subsequently disassembled). Interestingly, most of the incompletely synapsed bivalents had one end unsynapsed (i.e., a forked structure), and this was nearly always (93%) the centromeric end (Figure 3L and Table 2). A minority of bivalents had other synaptic anomalies such as having both ends unsynapsed or only interstitial asynapsis (a “bubble”) (Figure 3L and Table 2). The unsynapsed regions accounted for 28.2% of total axis length in a representative population of pachytene-like cells (N = 7 cells). Similar synaptic anomalies were also observed in *Trip13^sev/sev^* oocytes. Most oocyte bivalents at the pachytene-like stage presented some degree of asynapsis, with most showing unsynapsed centromeric ends (Figure 4 and Table 2).

**Table 2:** Synaptic anomalies in *Trip13^sev/sev^* spermatocytes and oocytes.

Synaptic anomaly	Number of aberrant bivalents in spermatocytes (375 bivalents total)	Number of aberrant bivalents in oocytes (136 bivalents total)
All anomalies	283 (75.4%)	100 (73.6%)
One end unsynapsed (fork)^a^	230 (61.3%)	89 (65.5%)
Only the centromeric end unsynapsed^a^	213 (56.8%)	87 (64.0%)
Both ends unsynapsed^a^	32 (8.5%)	5 (3.7%)
Interstitial asynapsis only (bubble)	21 (5.6%)	6 (4.4%)

a Irrespective of whether interstitial asynapsis was also present.

**Figure 5:**
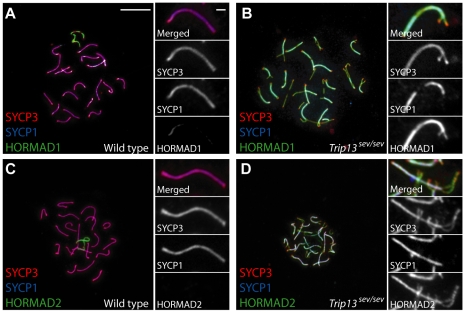
Depletion of HORMAD proteins from synapsed axes requires TRIP13. Spread chromosomes from pachytene wild-type spermatocytes (A, C) or pachytene-like *Trip13^sev/sev^* spermatocytes (B, D) were stained for SYCP3 (red), SYCP1 (blue), and in green for either HORMAD1 (A, B) or HORMAD2 (C, D). Previous studies demonstrated that HORMAD1 and HORMAD2 show continuous staining of unsynapsed chromosome axes in leptonema and zygonema and become substantially depleted from axes soon after synapsis occurs, and that this synapsis-associated HORMAD depletion does not occur properly in *Trip13^mod/mod^* spermatocytes [20]. *Trip13^sev/sev^* mutants show an indistinguishable defect, with both HORMADs remaining at high levels on synapsed axes. Bar in panel A applies to all main panels and represents 10 µm; bar in the inset to panel A applies to all insets and represents 1 µm.

Recent studies have demonstrated that *Trip13* is also required for changes in the protein composition of chromosome axes that accompany SC formation [20]. HORMAD1 and HORMAD2 are mammalian members of an evolutionarily conserved family of meiotic axis proteins, related to Hop1 in yeast [20], [21]. In mouse, HORMAD1 and -2 become substantially depleted from chromosome axes soon after synapsis in wild type, but not in *Trip13^mod/mod^* cells [20]. *Trip13^sev/sev^* mutants have a similar defect, with substantial levels of HORMAD1 and -2 staining retained on the axes of nearly fully synapsed chromosomes in pachytene-like cells (Figure 5).

### TRIP13 is required for XY synapsis and sex body formation

We also evaluated the effect of the *Trip13* mutations on the sex chromosomes. In pachytene spermatocytes, the X–Y pair forms only a short stretch of SC encompassing the small region of homology they share with one another, termed the pseudoautosomal region (PAR) (Figure 3C and 3G). Because the PAR is small (only ∼700 kb in at least one mouse strain [22]), this chromosome pair is very sensitive to perturbations of the synaptic process, and thus can report on subtle defects that might not be seen on the autosomes [23]. In *Trip13^mod/mod^* animals, the X and Y were separated at pachynema in 6.5% of spermatocytes (N = 154), a 7.2-fold increase over the frequency in wild type (0.9%, N = 116, p = 0.01 Fisher's exact test). Moreover, 54.2% of pachytene-like *Trip13^sev/sev^* spermatocytes displayed unsynapsed X and Y chromosomes (N = 24) (Figure 3K), an 8-fold increase over the frequency in *Trip13^mod/mod^* and a 60-fold increase over wild type. The stronger phenotype in *Trip13^sev/sev^* cells reinforces the significance of the modest XY synapsis defect found in *Trip13^mod/mod^* mutants.

**Figure 6:**
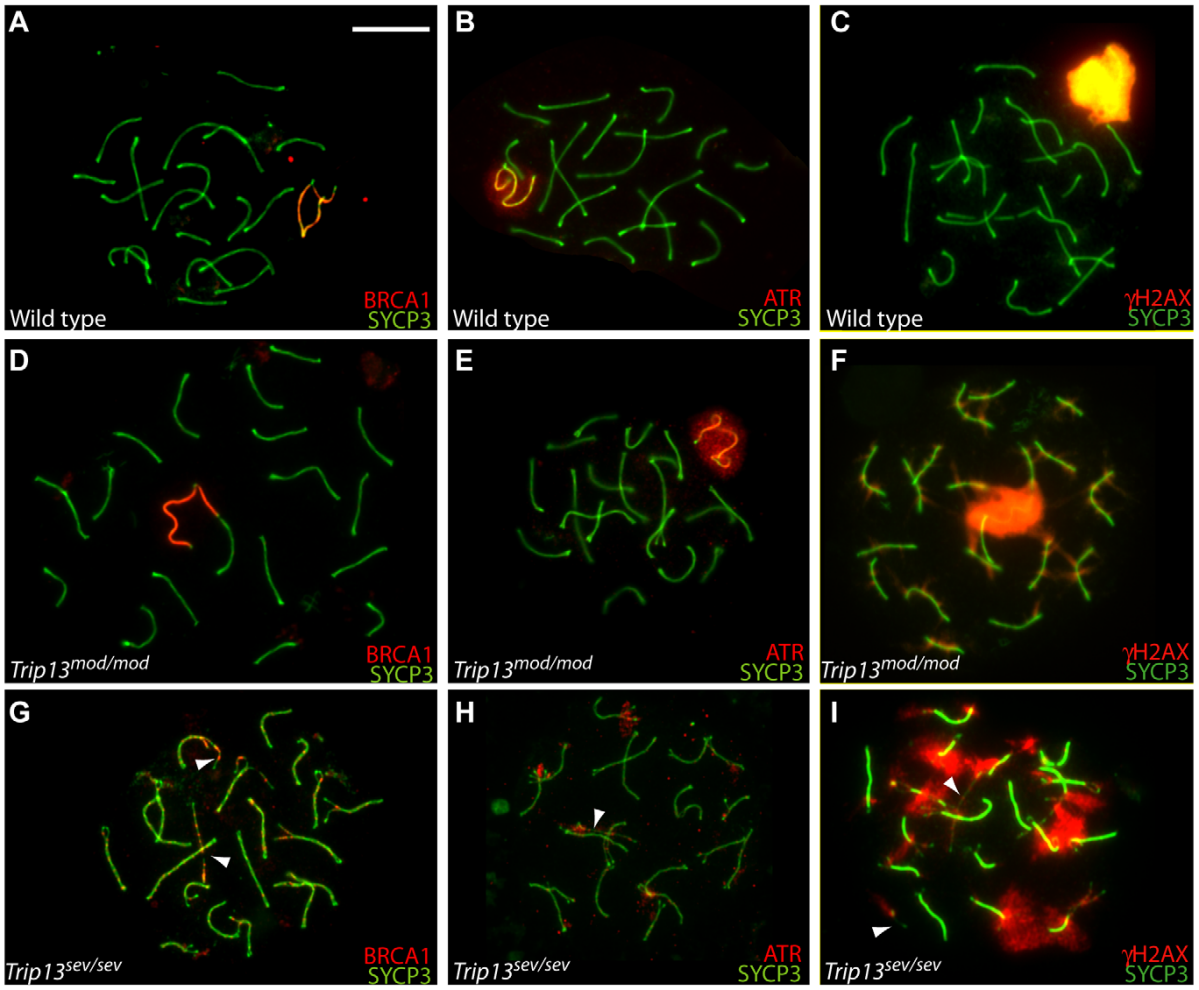
Sex body formation in *Trip13* mutant mice. Spread chromosomes of pachytene (or pachytene-like) spermatocytes from the indicated genotypes were stained with antibodies to SYCP3 and to either BRCA1 (A,D,G), ATR (B,E,H), or γH2AX (C,F,I) to monitor sex body formation. Arrowheads point to the sex chromosomes; in (G, H, I) identities of the X and Y are deduced from axis lengths and synaptic status (i.e., fully unsynapsed). Note that many of the synapsed autosomes show abnormal presence of BRCA1 or ATR foci in the *Trip13^sev/sev^* mutant (G, H) and of γH2AX in both mutants (F, I). Bar = 10 µm.

During prophase in spermatocytes, the X and Y form a sub-nuclear domain (the sex body) that is transcriptionally silenced (meiotic sex chromosome inactivation, MSCI) (reviewed in [24]). MSCI entails localization of BRCA1 protein to unsynapsed X and Y axes, subsequent recruitment of the kinase ATR to the axes and peripheral chromatin loops, and phosphorylation of the histone variant H2AX to form γH2AX, which appears on the X and Y chromatin from zygonema onwards (Figure 6A–6C). All pachytene *Trip13^mod/mod^* spermatocytes analyzed (N≥42 cells for each MSCI marker) showed apparently normal accumulation of BRCA1 and ATR on sex chromosomes and sex body formation as judged by staining for γH2AX (Figure 6D–6F), which indicates that MSCI takes place in *Trip13^mod/mod^* mutants. Normal sex body formation was also inferred from γH2AX patterns in earlier studies [16].

In contrast, only 32.0% of pachytene-like *Trip13^sev/sev^* spermatocytes (N = 100) showed X and Y chromosome axes coated with BRCA1, and only 0.7% of mutant cells (N = 141) had the normal pattern of ATR covering the X and Y axes and chromatin. Instead, most cells showed numerous BRCA1 or ATR patches or foci distributed on many chromosomes, with no clear preference for the X and Y (Figure 6G and 6H). These findings indicate that BRCA1 enrichment on the X and Y is not sufficient to ensure ATR enrichment. Only 9.8% of pachytene-like *Trip13^sev/sev^* spermatocytes formed a sex body-like accumulation of γH2AX (N = 61), with most cells displaying prominent patches of γH2AX that did not co-localize with the sex chromosomes (Figure 6I). From analysis of other mouse mutants, it has been hypothesized that unsynapsed autosomal regions cause sex body failure by sequestering proteins required for MSCI [25]. It is thus likely that MSCI failure in *Trip13^sev/sev^* spermatocytes is an indirect consequence of autosomal asynapsis. MSCI failure is sufficient to trigger apoptosis of pachytene spermatocytes [24], [26], so this defect likely explains spermatogenic arrest in *Trip13^sev/sev^* mutants (but not in *Trip13^mod/mod^* mutants, since these are proficient for MSCI; see next section for further discussion).

### TRIP13 is required for timely completion of meiotic recombination

**Figure 7:**
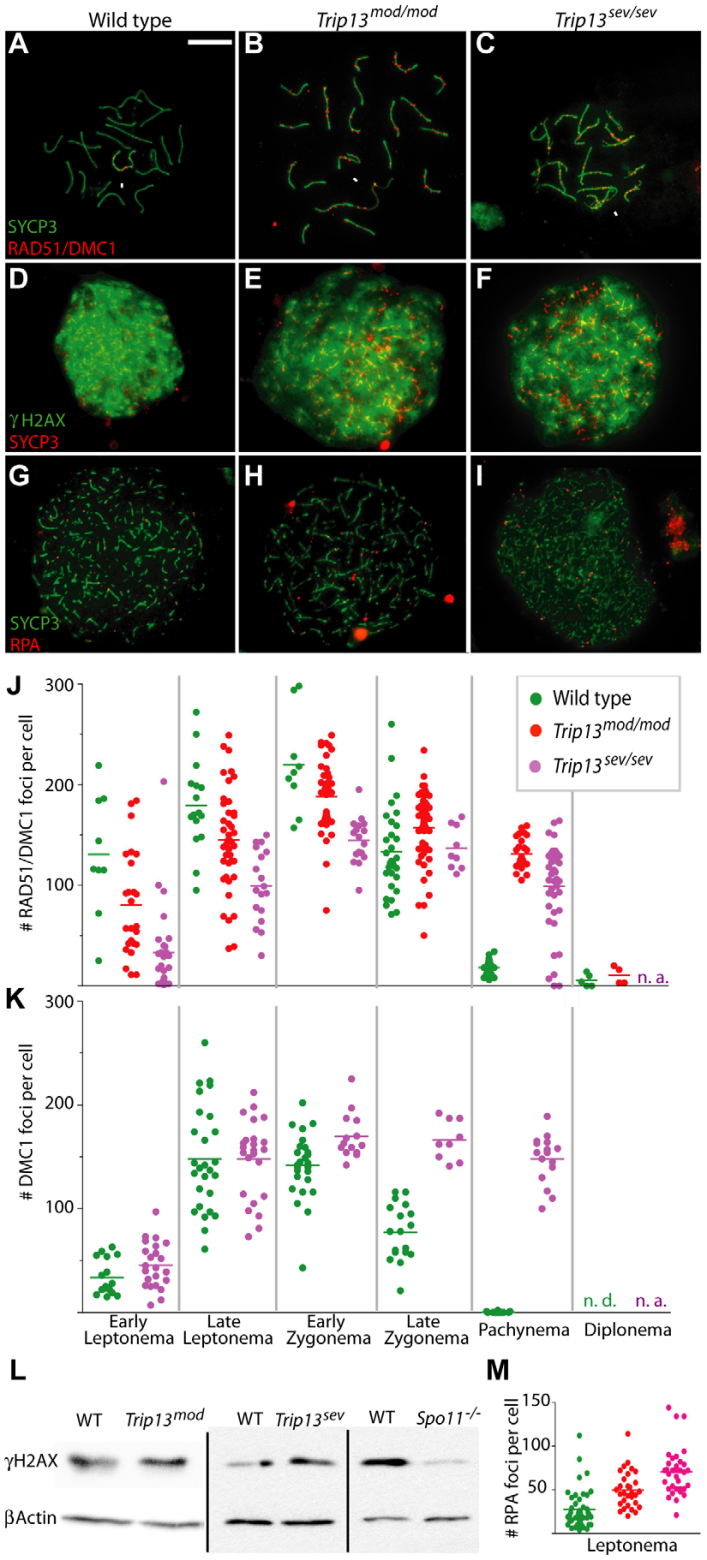
Early and late recombination defects in *Trip13* mutant spermatocytes. (A–C) Representative pachytene and pachytene-like nuclei of the indicated genotypes stained with anti-SYCP3 (green) and an anti-RAD51 antibody that also cross-reacts with DMC1 (red). Arrows indicate the sex chromosomes. Bar in (A) applies to all micrographs and represents 10 µm. (D–F) Representative leptotene nuclei stained for γH2AX (green) and SYCP3 (red). (G–I) Representative leptotene nuclei stained for SYCP3 (green) and RPA (red). (J, K) Quantification of the numbers of RAD51/DMC1 foci (detected with the cross-reacting anti-RAD51 antibody) or DMC1-specific foci per cell at the indicated stages. Horizontal lines denote the means; n.a., not applicable. See Table 3 for summary of means, standard deviations, and results of statistical tests. (L) Western blots to determine γH2AX levels in extracts from whole testes from juvenile males at 11.5 dpp. At this age, the majority of the γH2AX is in response to SPO11-generated DSBs; the residual γH2AX in *Spo11^–/–^* mutants is likely from somatic cells or premeiotic germ cells undergoing DNA replication [27]. For each mutant genotype, testes were also collected from a wild-type littermate. Blots were stripped and reprobed for β-actin as a loading control. (M) Quantification of numbers of RPA foci per cell in wild type, *Trip13^mod/mod^* and *Trip13^sev/sev^* spermatocytes [color code as in (J)].

As described in the Introduction, yeast Pch2 is needed for normal progression of homologous recombination toward both CO and NCO outcomes, and previous work provided evidence that mouse TRIP13 is also required for completion of recombination [16]. The availability of two *Trip13* mutant alleles with different severity provided an opportunity to examine this issue in greater detail. DSB repair can be followed indirectly by staining chromosomes for γH2AX, which forms rapidly on chromatin near DSBs [27], and for strand-exchange proteins RAD51 and DMC1, which form cytological complexes at sites of ongoing recombination [28], [29]. During zygonema, the number of RAD51/DMC1 foci peaks and γH2AX progressively disappears where synapsis has occurred. In normal pachynema, most of these early DSB markers have disappeared from autosomes and only a handful of RAD51/DMC1 foci remain on the unsynapsed regions of sex chromosomes (Figure 7A), along with the DSB-independent γH2AX in the sex body (Figure 6C).

During pachynema, as previously described [16], *Trip13^mod/mod^* mice showed persistent γH2AX and RAD51/DMC1 foci on synapsed autosomes, an indicator of delayed and/or inefficient DSB repair (Figure 6F and Figure 7B). In our analysis, *Trip13^mod/mod^* spermatocytes at this stage displayed 7.2-fold more RAD51/DMC1 foci than wild type (Figure 7J and Table 3). Similarly, pachytene-like *Trip13^sev/sev^* spermatocytes showed persistent γH2AX on unsynapsed autosomes (Figure 6I), and RAD51/DMC1 foci were also substantially elevated, although lower on average and more variable than in the weaker mutant (5.4-fold higher than wild type; Figure 7C and 7J and Table 3). The few *Trip13^mod/mod^* cells that successfully reached diplonema had normal (i.e., very low) levels of RAD51/DMC1 foci (Figure 7J and Table 3), so it is likely that these represent a small subset of cells that successfully repaired most of their DSBs due to residual expression of TRIP13. No diplotene cells were recovered from *Trip13^sev/sev^* testes. The anti-RAD51 antibody used for this analysis shows weak cross-reaction with DMC1 on western blots (data not shown), so we also examined wild-type and *Trip13^sev/sev^* nuclei immunostained with a DMC1-specific antibody. In wild-type pachynema, essentially all DMC1 foci have disappeared, but pachytene-like *Trip13^sev/sev^* spermatocytes showed large numbers of persistent DMC1 foci (>300-fold more than wild-type littermates; Figure 7K and Table 3).

**Table 3:** Numbers of early recombination-associated foci in spermatocytes.

		Genotype		
Protein	Stage	Wild type	*Trip13^mod/mod^*	*Trip13^sev/sev^*
RPA	Leptonema	27.6±22.0	52.1±20.9^a^	70.2±26.9a,b
RAD51/DMC1^c^	Early Leptonema	130.7±60.4	82.3±52.0^a^	33.3±43.3a,b
	Late Leptonema	179.3±45.3	146.9±50.4^a^	99.5±35.5a,b
	Early Zygonema	219.7±49.1	189.9±36.4^a^	144.5±22.7a,b
	Late Zygonema	133.3±43.7	156.3±34.8^a^	136.9±21.6
	Pachynema	18.2±7.0	131.0±14.5^a^	99.0±42.6a,b
	Diplonema	6.0±6.2	13.0±8.9	n.a.
DMC1^d^	Early Leptonema	33.7±17.0	n.d.	45.5±22.2
	Late Leptonema	147.8±50.7	n.d.	147.8±38.1
	Early Zygonema	141.8±31.1	n.d.	169.7±21.9^a^
	Late Zygonema	77.2±27.0	n.d.	166.0±19.2^a^
	Pachynema	0.4±0.7	n.d.	147.8±23.5^a^

a Significantly different from wild type (p≤0.05, t test).

b Significantly different from *Trip13^mod/mod^* (p≤0.05, t test).

c Detected with an anti-RAD51 antibody that cross-reacts with DMC1.

d Detected with a DMC1-specific antibody.

n.a., not applicable; n.d., not determined.

Analyses of other cytological markers extend these findings. Specifically, 8% of *Trip13^mod/mod^* pachytene spermatocytes displayed discrete BRCA1 foci on synapsed autosomes (N = 50), which was never observed in wild type (N = 58) (data not shown). In contrast, such foci were observed in 73% of pachytene-like *Trip13^sev/sev^* spermatocytes (N = 100 cells) (Figure 6G). Pachytene-like *Trip13^sev/sev^* spermatocytes also displayed ATR foci on synapsed autosomes (Figure 6H), which were not observed in wild type or *Trip13^mod/mod^* (data not shown). These patterns indicate that *Trip13^sev/sev^* spermatocytes have a more severe block to recombination.

Previous studies of other mouse recombination mutants demonstrated that the presence of unrepaired DSBs in late zygotene and early pachytene spermatocytes provokes a regulatory response that is separable from MSCI failure alone [30]. Thus, it seems likely that *Trip13^mod/mod^* spermatocytes undergo apoptosis as a direct consequence of defects in repair of SPO11-induced DSBs (see also ref. [24]). In oocytes, meiotic DSB repair defects cause early and severe meiotic arrest that is partially suppressed by eliminating DSB formation [19]. *Trip13* mutant ovaries resemble those of DSB repair-defective mutants such as *Dmc1^–/–^* and *Msh5^–/–^* (Figure 2H and ref. [19]), and mutation of *Spo11* substantially suppresses the early oocyte loss phenotype of *Trip13^mod/mod^* animals [16], strongly suggesting that unrepaired breaks lead to oocyte loss in *Trip13* mutants.

### TRIP13 is required early in DSB repair

The above findings support the conclusion that TRIP13 is needed for timely and efficient completion of meiotic recombination. However, further analysis revealed informative defects even earlier in meiotic prophase. From early leptonema to early zygonema, *Trip13^mod/mod^* spermatocytes had significantly fewer RAD51/DMC1 foci than wild-type cells of equivalent stage (63–86% of wild type on average, depending on stage; Figure 7J and Table 3). *Trip13^sev/sev^* spermatocytes displayed even lower numbers (25–66% of wild type; Figure 7J and Table 3). Staining with the DMC1-specific antibody revealed a very different result, however: strikingly, there was no detectable difference in numbers of DMC1 foci between wild type and *Trip13^sev/sev^* in early or late leptonema (Figure 7K and Table 3). These findings strongly suggest that TRIP13 is required specifically for assembly of normal numbers of RAD51 complexes. RAD51 and DMC1 colocalize extensively, such that most recombination-associated foci stain positive for both proteins [31]. In wild-type, there are fewer DMC1 foci than RAD51 foci (Figure 7J and 7K); assuming equivalent detection efficiencies, this finding suggests that there are a subset of foci that contain predominantly RAD51. These could be a distinct subset of DSB sites that never accumulate DMC1 or, alternatively, a reflection of changes in protein composition over time at recombination sites. In any case, we note that numbers of DMC1 foci throughout prophase in *Trip13^sev/sev^* are sufficient to account for all of the foci observed with the less specific anti-RAD51 antibody (Table 3). (The lower numbers of anti-RAD51 foci may reflect lower efficiency for detection of DMC1 by this antibody, as suggested by weaker anti-DMC1 western blot signal (data not shown).) It is possible that little or no RAD51 is ever loaded at DSB sites in this mutant, or that the mutant is specifically defective for forming a subpopulation of RAD51-only foci. More highly specific anti-RAD51 reagents will be necessary to evaluate this issue fully.

Observing fewer RAD51 foci at early stages was unexpected, but the greater severity of *Trip13^sev/sev^* mutants reinforces the significance of this finding. The normal numbers of DMC1 foci in leptonema in *Trip13^sev/sev^* indicate that DSB formation is neither reduced nor delayed, but to address this question more thoroughly, we examined γH2AX formation during leptonema, which is provoked by SPO11-generated DSBs in a largely ATM-dependent fashion [23], [27], [32]. Both *Trip13* mutants showed γH2AX distributed widely across the chromatin in leptonema, with timing and spatial patterns comparable to those seen in wild type (Figure 7D–7F). To compare amounts of SPO11-induced γH2AX, we carried out western blotting on extracts from testes of juvenile mice, in which the only spermatocytes present are in the first, semi-synchronous wave of meiosis. At 11.5 dpp, most spermatocytes are in leptonema or zygonema, and few, if any, cells have reached later stages when MSCI-associated γH2AX formation begins (data not shown). Thus, the majority of the γH2AX signal at this age is SPO11-dependent (compare wild type with *Spo11^–/–^* littermates in Figure 7L, right panel). In this analysis, *Trip13^mod/mod^* and *Trip13^sev/sev^* mutants displayed similar γH2AX levels as wild-type littermates (Figure 7L). These findings strongly indicate that DSBs form with normal timing and in roughly normal amounts in both *Trip13* mutants.

We also examined chromatin-bound complexes of the ssDNA binding protein RPA, which acts early in recombination to enhance formation of RAD51 and DMC1 nucleoprotein filaments on ssDNA [33]. Normal leptotene spermatocytes display relatively few RPA foci despite the presence of numerous DSBs with ssDNA tails, presumably because RPA is rapidly displaced by RAD51 and DMC1 [34]. (Abundant, prominent foci of RPA are observed later on synapsed chromosome axes after RAD51/DMC1 complexes have disappeared [35], but these RPA foci most likely mark D-loops or other later recombination intermediates.) If TRIP13 promotes the assembly of RAD51 onto resected DSB ends, we reasoned that RPA foci might accumulate to higher numbers in leptotene spermatocytes in the *Trip13* mutants. Indeed, RPA foci were increased 1.9-fold (*Trip13^mod/mod^*) and 2.5-fold (*Trip13^sev/sev^*) relative to wild type (Figure 7G–7I and 7M) (p≤0.0012 for all pair wise comparisons, t test). Taken together, our findings place TRIP13 function earlier in recombination than previously anticipated, before RAD51 focus formation but after formation and resection of SPO11-induced DSBs.

### TRIP13 is needed for normal CO numbers and to ensure that at least one chiasma forms per bivalent

#### Autosomes

Only a subset of DSBs are repaired as COs. The remainder, ∼90% in mouse, are presumed to be repaired as NCOs. Because *Trip13^mod/mod^* pachytene spermatocytes appeared to have numbers of MLH1 foci (a marker for COs) similar to values typically reported for wild type, it was hypothesized that the persistent DSBs in *Trip13^mod/mod^* spermatocytes reflect a NCO-specific defect [16]. However, because *pch2* mutant yeast have abnormal CO patterns, we examined this issue in more detail. As detailed below, we find that TRIP13 is required for normal CO numbers in both male and female meiosis and, moreover, that TRIP13 is needed for formation of at least one CO/chiasma per chromosome pair.

**Figure 8:**
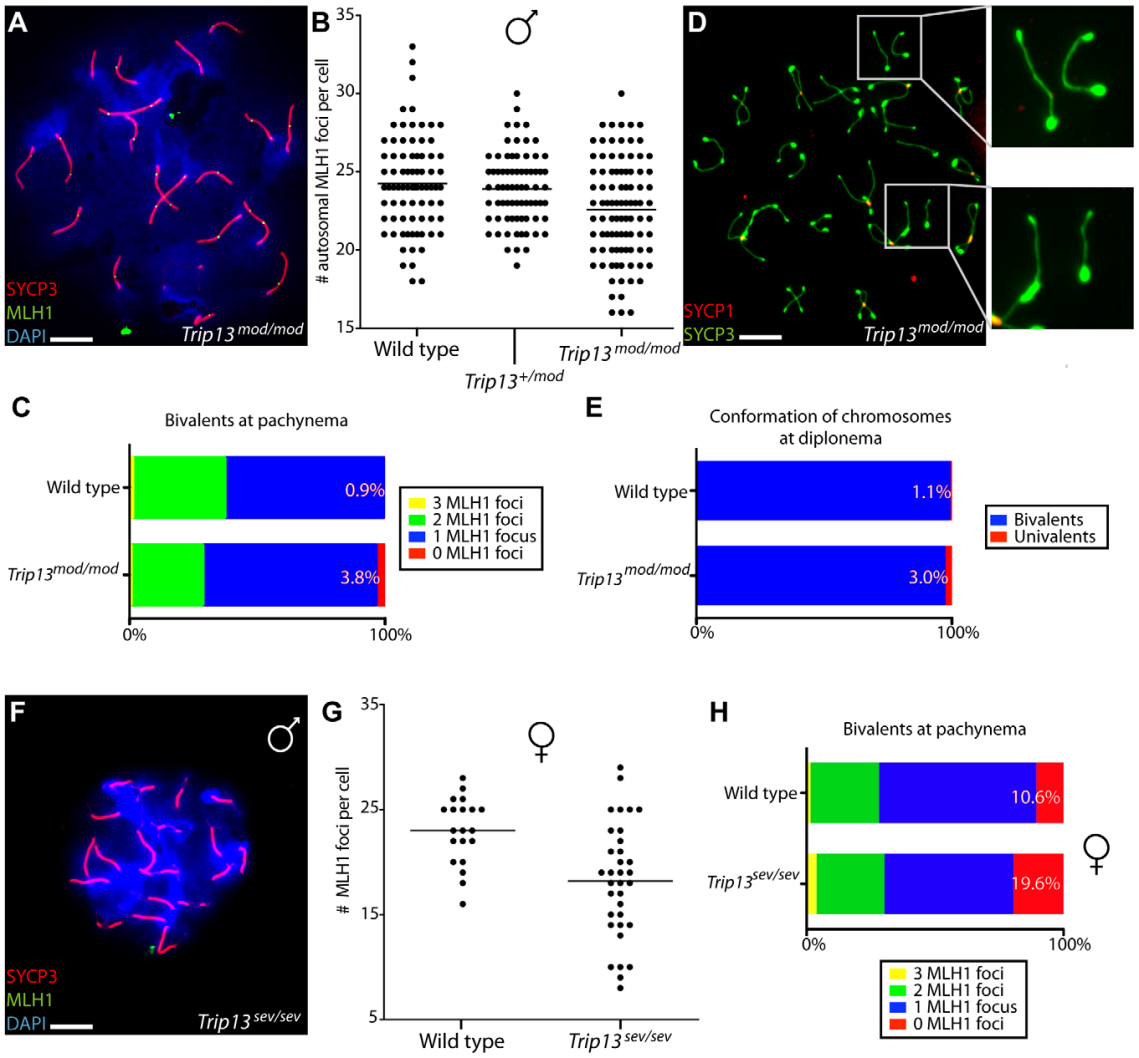
Reduced COs and chiasmata in *Trip13*-deficient spermatocytes and oocytes. (A) Spread chromosomes from a *Trip13^mod/mod^* pachytene spermatocyte stained for DNA (DAPI), SYCP3, and MLH1 to mark the positions of COs. Bar = 10 µm. (B) Quantification of the number of autosomal MLH1 foci per pachytene cell. Means are shown as horizontal lines. Results were as follows (mean ± sd): 24.2±3.1 (wild type, six mice); 23.9±2.3 (*Trip13^+/mod^*, seven mice); 22.6±3.2 (*Trip13^mod/mod^*, four mice). (C) Percentages of bivalents in wild-type and *Trip13^mod/mod^* pachytene spermatocytes with the indicated numbers of MLH1 foci. (D) Spread chromosomes from a *Trip13^mod/mod^* diplotene spermatocyte stained for SYCP3 and SYCP1. Note the presence of achiasmate bivalents, some of which are presented in the zoomed images on the right. Bar = 10 µm. (E) Percentages of achiasmate chromosome pairs at diplonema in wild-type and *Trip13^mod/mod^* spermatocytes. (F) Absence of MLH1 foci in a pachytene-like *Trip13^sev/sev^* spermatocyte. Bar = 10 µm. (G) Quantification of MLH1 focus numbers in pachytene or pachytene-like oocytes. Means are indicated by the horizontal line. Results were as follows (mean ± sd): 23.0±3.2 (wild type); 18.2±5.5 (*Trip13^sev/sev^*). (H) Percentages of bivalents with the indicated numbers of MLH1 foci. This analysis provides a conservative estimate of the defect in *Trip13^sev/sev^* oocytes, because only cells with ≥20 total foci were considered in order to minimize possible secondary effects of differences in the timing of progression through meiosis.

In agreement with prior studies, we readily observed autosomal MLH1 foci in *Trip13^mod/mod^* spermatocytes (Figure 8A). Surprisingly, however, there was a small but significant reduction in the total number per cell when compared to wild-type littermates (Figure 8B; p = 0.002, Mann-Whitney test). *Trip13^+/mod^* mice were indistinguishable from their wild-type littermates (Figure 8B; p = 0.44). There was a significant decrease in the number of bivalents with two MLH1 foci (28.1% of bivalents in *Trip13^mod/mod^* compared to 36.1% in wild type), and a significant increase in the number of bivalents with no detectable focus (3.8% in the mutant (N = 420 bivalents) compared to only 0.9% in wild-type spermatocytes (N = 332), p = 0.017, G test) (Figure 8C).

If the reduced MLH1 foci in *Trip13^mod/mod^* cells reflect a decrease in COs rather than a difference in ability to detect COs via MLH1 staining, then we also expect an increase in the number of achiasmate chromosomes (univalents) at diplonema. Consistent with this expectation, 3.0% of chromosome pairs in *Trip13^mod/mod^* cells were achiasmate compared to 1.1% in wild type (Figure 8D and 8E) (N = 1808 and 1083 bivalents, respectively; p = 0.0008, Fisher's exact test), similar to the frequency of pachytene bivalents lacking an MLH1 focus (3.8%). Interestingly, most of the separated univalents at diplonema (77.3%, N = 22 pairs) remained parallel in the spread nuclei (see insets in Figure 8D for examples), implying that some sort of physical connections persist between the homologs after SC disassembly. These connections might be COs that failed to mature into chiasmata (and that would perhaps have failed to accumulate MLH1). Alternatively, DNA catenation might hold the paired homologs together during the chromosome spreading process.

MLH1 foci were not detectable in pachytene-like spermatocytes from *Trip13^sev/sev^* mice (Figure 8F). This finding could mean that TRIP13 is essential for CO formation per se, but a more likely possibility is that mutant spermatocytes never reach the stage at which MLH1 foci appear (mid to late pachynema), because other mutants that fail to form a proper sex body arrest prior to mid-pachynema [e.g. [25],[30]]. To circumvent this complication, we investigated MLH1 focus formation in *Trip13^sev/sev^* oocytes, which do not normally carry out MSCI and which thus do not show the same pachytene arrest as spermatocytes in response to MSCI-defective mutations [36]. MLH1 foci were readily detected in *Trip13^sev/sev^* oocytes at pachynema (data not shown), indicating that TRIP13 is not essential for CO formation. However, *Trip13^sev/sev^* oocytes had fewer MLH1 foci than wild-type littermates (Figure 8G, p≤0.001, Mann-Whitney test), similar to but more severe than the situation in *Trip13^mod/mod^* spermatocytes. This reduction is mainly attributable to an increase in the number of bivalents lacking an MLH1 focus (19.5% of bivalents in *Trip13^sev/sev^* (N = 231), vs. 10.6% in wild type (N = 273), p = 0.0055, Fisher's exact test) (Figure 8H).

#### The XY pair

At diplonema, 14.5% of *Trip13^mod/mod^* spermatocytes (N = 93) had achiasmate X and Y chromosomes, compared with 9.7% of wild-type cells (N = 31). This difference is not statistically significant (p = 0.7492, Fisher's exact test), although we do note that the difference in frequency (i.e., 4.8% higher in *Trip13^mod/mod^*) is of comparable magnitude to the difference in frequency of asynaptic XY pairs at pachynema (5.6% higher; see above). Thus, it may be the case that *Trip13^mod/mod^* spermatocytes have a modest XY chiasma defect comparable to the earlier XY synapsis defect, but larger sample sizes would be required to detect a difference this small. Importantly, this result suggests that, for sex chromosomes that successfully pair and synapse, subsequent chiasma formation is not substantially worse than for autosomes (see Discussion).

### Cytological interference is altered in TRIP13-deficient spermatocytes

**Figure 9:**
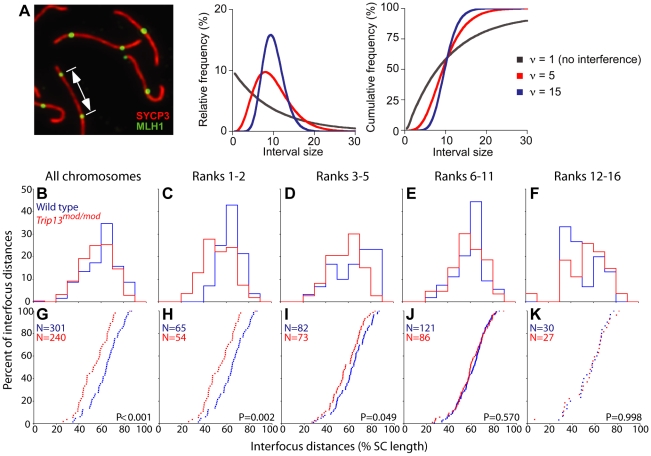
Reduced MLH1 inter-focus distances in *Trip13^mod/mod^* spermatocytes. (A) Pachytene spermatocyte spreads were immunostained for SYCP3 (shown in red) and MLH1 (shown in green), then the distances between foci were measured on autosomal bivalents that contain two or more MLH1 foci. Examples of relative and corresponding cumulative frequency plots of gamma distributions are shown. If there is no interference between MLH1 foci, an exponential frequency distribution is expected (gray lines). Deviation from exponential behavior indicates the existence of interference (red and blue lines): short and long distances become more rare and the spacing becomes more even (i.e., distances are tightly clustered). Curves were calculated using an average interfocus distance of 10 and the indicated increasing values for the shape parameter, **υ**. Adapted from ref. [23]. (B–K) Inter-focus distances in wild type and *Trip13^mod/mod^* spermatocytes. (B–F) show the frequency distributions (step plots) of inter-focus distances as a percentage of the SC length for wild type (blue) and *Trip13^mod/mod^* (red). (G–K) show cumulative plots of the same data, with the number of chromosomes analyzed and the Kolmogorov-Smirnov test p value indicated. The left column of graphs (B, G) pools data for all autosomes. For the remaining columns, the autosomal bivalents in each nucleus were rank-ordered by SC length from largest (1) to smallest (19), then divided into groups of similarly sized chromosomes: the two largest chromosomes are shown in (C, H), the next three in (D, I), and so forth. Autosome size ranks 17–19 are excluded from this analysis because they rarely have more than a single MLH1 focus.

In most organisms, the presence of a meiotic CO in one genomic region makes it less likely that another CO will be found nearby [37]. This phenomenon, called CO interference, results in a wider and more even spacing of COs than expected if they were distributed randomly relative to one another [38], [39]. Direct measurement of CO interference is difficult in mice, and is currently impossible in sterile mouse mutants. However, interference can be measured cytologically by analyzing the distances separating pairs of adjacent MLH1 foci on pachytene chromosomes (Figure 9A) [38], [40]. When many such distances are examined, the strong interference typical of mammalian meiosis is readily seen: the relatively even spacing of MLH1 foci manifests as tight clustering of interfocus distances in frequency distribution plots (Figure 9B–9F, blue lines) and the steeply sigmoidal shape of cumulative frequency plots of the same data (Figure 9G–9K). Wide spacing is seen from the rarity of MLH1 focus pairs that are separated by short distances (e.g., only 0.3% of focus pairs were separated by ≤25% of the length of the bivalent) (Figure 9B and 9G).

Substantial interference was also observed in *Trip13^mod/mod^* spermatocytes, as revealed by a relatively narrow distribution of interfocus distances and by the rarity of closely spaced foci (0.4% of focus pairs were separated by ≤25% of the bivalent length) (red in Figure 9B and 9G). A routinely used approach to quantifying cytological interference is to model MLH1 inter-focus distances to a gamma distribution [23], [38]. The gamma distribution that best fits the observed frequency distribution is characterized by a shape parameter (abbreviated “**υ**”), which can be regarded as a measure of the strength of interference between MLH1 foci: a value of **υ** = 1 implies no interference, whereas higher **υ** values indicate more regular spacing between foci, and thus stronger interference (Figure 9A). There appears to be a trend toward lower **υ** values in the *Trip13^mod/mod^* mutant (Table 4), which may indicate that MLH1 foci are less evenly spaced than in normal meiosis. However, this analysis confirms that substantial cytological interference is retained in the mutant.

**Table 4:** Gamma distribution parameters for MLH1 inter-focus distances.

Genotype	Chromosome rank	No. of intervals	υ (corrected)^a^
Wild type	All chromosomes	301	10.9
	1–2	65	20.1
	3–5	82	15.2
	6–11	121	17.0
	12–16	30	7.9
*Trip13^+/mod^*	All chromosomes	246	12.6
	1–2	52	21.2
	3–5	70	22.4
	6–11	97	13.6
	12–16	26	5.4
*Trip13^mod/mod^*	All chromosomes	240	9.2
	1–2	54	14.1
	3–5	73	14.0
	6–11	86	11.9
	12–16	27	4.1

a Best-fit gamma distribution shape parameters, corrected as described [38] for the fact that the gamma distribution assumes theoretical limits of infinitely small and infinitely large inter-focus distances, but the actual range of inter-focus distances that can be detected is limited by the resolution of light microscopy and by the finite length of each SC.

Nonetheless, closer inspection revealed an intriguing difference, namely, that there was a significant decrease in the average separation between MLH1 foci, from 60.8±13.4% of bivalent length in wild type to 56.8±13.8% in the mutant (mean ± sd; p<0.0007, two-sided t-test) (Figure 9B and 9G). Mean MLH1 focus separation in *Trip13^+/mod^* heterozygotes was indistinguishable from wild type (60.9±13.4%; data not shown). Expressing these measurements as percent of bivalent length corrects for the slightly shorter SCs in *Trip13^mod/mod^* cells (see above); the shift is much more pronounced if data are not normalized (Figure S1). Because different chromosomes show reproducible differences in CO distributions [e.g., [38],[41]], we further analyzed these data after dividing into groups of similarly sized chromosomes. Strikingly, the shift toward shorter interfocus distances was specific for the largest chromosomes (size ranks 1–5; Figure 9C, 9D, 9H, 9I). Thus, the altered interfocus distances are not a trivial consequence of the shorter SCs, because chromosomes of all size classes were affected equally by the decrease in SC length (Table 1).

### CO distributions along chromosomes are altered in TRIP13-deficient spermatocytes

COs show biased distributions along chromosomes (reviewed in [42]), which at least in part reflects position-specific differences in the likelihood that a given recombination intermediate will become a CO [38]. Few mouse mutants have been found that alter the CO distribution without blocking the recombination process outright, but as detailed below, *Trip13^mod/mod^* spermatocytes show pronounced changes in the distribution of MLH1 foci, implicating TRIP13 in the processes that regulate CO position.

**Figure 10:**
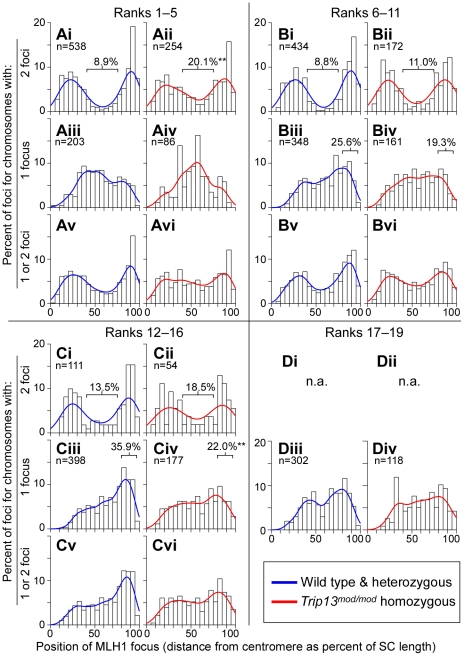
Altered CO distributions on autosomes in *Trip13*-deficient spermatocytes. Spread pachytene nuclei were stained for SYCP3, MLH1, and DAPI (to detect pericentromeric heterochromatin), and the positions of MLH1 foci were measured relative to centromeres and expressed as percentage of SC length. Because chromosomes of different size show different distributions of MLH1 patterns, data are presented for groups of similarly sized chromosomes, rank ordered as described in the legend to Figure 9. No significant differences were observed between wild type and *Trip13^+/mod^*, so data for these genotypes were pooled for simplicity; separated data are provided in Figure S2. Frequency distribution histograms are shown separately for MLH1 foci on chromosomes with exactly two foci (graphs i–ii in each panel), chromosomes with only one focus (graphs iii–iv), or for all MLH1 foci (graphs v–vi). Chromosomes with three foci were rare and are omitted from this analysis. For the smallest size ranks (D), bivalents with two foci were rare so these were also omitted (n.a., not applicable). Smoothed curves (Gaussian kernel density estimates) are overlaid to facilitate comparison (dark blue, wild type and *Trip13^+/mod^*; red, *Trip13^mod/mod^*). The number of foci in each category is indicated. The central regions (from 40–75% of bivalent length) are bracketed for chromosomes with two foci, and the percentages of foci within these regions are indicated. Telomere-proximal regions (80–95% of bivalent length) and the percentages of foci within them are indicated in panels Biii, Biv, Ciii, Civ. Asterisks indicate values significantly different from wild type (Fisher's exact test, p<0.05).

Distributions of MLH1 focus positions on pachytene spermatocyte autosomes are shown in Figure 10. Distances, measured along the SC from the centromere, are expressed as percent of SC length to normalize for the decreased length in *Trip13^mod/mod^* cells and to facilitate comparisons of different sized chromosomes. For all patterns discussed below, results were highly reproducible between wild type and *Trip13^+/mod^* heterozygotes (Figure S2; p = 0.51 for all chromosomes, Anderson-Darling two-sample test), so data for these genotypes were pooled for clarity.

Chromosomes show distinct MLH1 localization patterns depending on their size and whether a bivalent has one or two foci [38], [41], so similar-sized chromosomes were grouped for comparison. Normal cells show a number of characteristic features, as documented previously (e.g., refs. [40], [41]) (Figure 10, dark blue curves). First, MLH1 foci are rare in the centromere-proximal ∼15% of each bivalent. Second, chromosomes with two MLH1 foci tend to have those foci clustered into a proximal region and a distal region, with very few in between (Figure 10Ai, 10Bi, 10Ci), reflecting the wide spacing between COs caused by interference. Third, there is a greater chance that a focus will be centrally located when a chromosome has only a single MLH1 focus, although singleton foci can also occur in more proximal or more distal regions as well (Figure 10Aiii, 10Biii, 10Ciii, 10Diii). Finally, singleton MLH1 foci are more likely to be located distally on smaller chromosomes than on larger ones (e.g., compare Figure 10Aiii with Figure 10Ciii). Distal MLH1 localization appears to reflect a position-specific tendency for recombination events to favor (or not) a CO outcome [38] and, unlike patterns for chromosomes with two foci, cannot be accounted for simply as a consequence of wide spacing imposed by interference.

In *Trip13^mod/mod^* spermatocytes, CO repression near centromeres was intact, as inferred from the rarity of MLH1 foci within the first 15% of each chromosome (43 of 1021 foci in *Trip13^mod/mod^* (4.2%), compared with 99/2311 in wild type and *Trip13^+/mod^* (4.3%)) However, other patterns were altered such that the normal biases appeared relaxed (Figure 10, red curves, p = 0.0006, Anderson-Darling two-sample test). Larger chromosomes (size ranks 1–5) that had two foci showed less extreme biphasic clustering in the mutant, with foci occurring 2.3-fold more frequently than wild type in the central regions of the chromosomes (Figure 10Aii, p = 1.4×10^−5^, Fisher's exact test). Mid-size chromosomes (ranks 6–11 and 12–16) showed the same tendency, but differences were not statistically significant (Figure 10Bii and 10Cii, p≥0.23). Importantly, the fact that this alteration is seen on chromosomes with two foci argues against the possibility that distributions in the mutant are solely a consequence of a shift toward more chromosomes in the population having only one focus.

In addition, chromosomes in size ranks 12–16 with a single focus were more likely to have that focus in the central and proximal regions in *Trip13^mod/mod^* cells, at the expense of the normal preference for distally positioned foci (Figure 10Ciii and 10Civ, p = 0.0006). Chromosomes in size ranks 6–11 showed a similar pattern, but this difference was not statistically significant (Figure 10Biii and 10Biv, p = 0.07). The net effect of these alterations is that MLH1 foci, and presumably COs, are more evenly (i.e., randomly) distributed. Importantly, all size classes and both one-focus and two-foci chromosomes showed similar overall tendencies, albeit manifesting more markedly on some chromosomes than others.

## Discussion

Budding yeast Pch2 has emerged from recent studies as a factor that promotes normal recombination patterns and other aspects of chromosome dynamics during normal meiosis, in addition to its initially identified role as a checkpoint factor. The mouse Pch2 ortholog, TRIP13, is required for completion of meiotic recombination, but specific details of the *Trip13^mod/mod^* phenotype suggested that functions of TRIP13 in mouse are very different from functions of Pch2 in yeast. In this work, we uncover new meiotic roles for TRIP13, taking advantage of two *Trip13* mutations with different severity. TRIP13-deficient meiocytes display hallmarks of both early and late recombination defects: reduced RAD51 focus formation, inefficient completion of recombination, reduced numbers of COs and chiasmata, and altered CO distributions. These defects provide the first evidence for an early function of TRIP13 in meiotic recombination and also implicate TRIP13 in recombination pathways that lead to both COs and NCOs. We also demonstrate that TRIP13 is required for completion of synapsis and for formation of structurally normal SC. Thus, TRIP13 is important both for normal recombination and for development of higher order chromosome structure in mouse, which is reminiscent of at least some Pch2 roles in yeast [9], [14], [15]. Although not all aspects of the mutant phenotypes are congruent in the two species, our results in conjunction with recent studies of HORMAD protein localization [20], suggest that there is greater conservation of TRIP13/Pch2 function than was previously apparent.

### TRIP13 is required at an early step(s) in meiotic recombination and is involved in recombination pathways leading to both COs and NCOs

Previous studies [16] and those reported here demonstrate that *Trip13^mod/mod^* meiocytes inefficiently repair meiotic DSBs, as evidenced by abnormal persistence of several cytological markers (e.g., DMC1 (and possibly RAD51), γH2AX, BRCA1, and ATR) into late meiotic prophase. Intriguingly, however, the *Trip13* mutants also display recombination defects much earlier in prophase, manifested as reduced numbers of foci that stain with an anti-RAD51 antibody during early leptonema through early zygonema. These findings are the first to situate TRIP13 function upstream of RAD51. In principle, the reduced focus numbers could mean that DSBs are reduced or delayed. However, in both *Trip13* mutants, DMC1 foci, RPA foci, and γH2AX appeared in early leptonema, i.e., with similar timing with respect to axial element formation as wild type. Moreover, γH2AX levels were similar between wild type and TRIP13-deficient cells, and RPA foci were more numerous in the mutants at leptonema. We therefore favor the interpretation that DSBs are generated and resected in relatively normal numbers and in a timely fashion in the mutants, and that TRIP13 is required (directly or indirectly) for efficient execution of recombination steps at or prior to loading of RAD51 onto resected DSB ends. DMC1 loading is not affected, however. Interestingly, *Brca1* mutant spermatocytes have also been reported to be specifically defective for assembly of RAD51 but not DMC1 foci [43], suggesting that TRIP13 function may be related to that of BRCA1. These results are in contrast to those with *Brca2* or *Tex15* mutant spermatocytes, which are defective for both RAD51 and DMC1 foci [34], [44], thus defining two classes of proteins that can be differentiated based on recombinase loading.

The numbers of DMC1 (and possibly RAD51) foci that persist in pachynema in the *Trip13* mutants (≥100 per cell) are in large excess over the number of recombination events that become COs in normal or *Trip13^mod/mod^* meiosis (∼20–25 per cell). The simplest interpretation is that most of the persistent foci represent sites of recombination intermediates that would have been resolved as NCOs (or intersister events) if they had been able to go to completion. Thus, a major function of TRIP13 is efficient execution of a NCO recombination pathway(s) [16]. However, close analysis revealed that *Trip13^mod/mod^* spermatocytes displayed fewer MLH1 foci at pachynema and more achiasmate autosome pairs at diplonema. The achiasmate frequency matched the frequency of pachytene autosomes that lacked an MLH1 focus, suggesting that this problem occurs even in those cells that escaped pachytene arrest because they had succeeded in repairing all of their DSBs. *Trip13^sev/sev^* pachytene oocytes also had fewer MLH1 foci. These findings suggest that TRIP13 is not absolutely required for CO or chiasma formation, but is needed for formation of wild-type numbers of COs and/or chiasmata as well as efficient formation of the obligate CO/chiasma. Importantly, these results reveal that the function of TRIP13 is not restricted solely to recombination pathways that lead to NCOs.

### TRIP13 influences crossover distributions

Cytological interference between MLH1 foci remains strong in *Trip13^mod/mod^* pachytene spermatocytes, but we observed a significant decrease in the average distance between pairs of adjacent MLH1 foci. This decrease was seen even when normalizing for decreased SC length, and was more pronounced for longer chromosomes even though all chromosomes showed equivalent reductions in SC length. Thus, it appears that the interference alteration is not readily explained as a trivial consequence of shortened SCs. It is thought that chromosome axes serve as the conduit for signals that mediate interference [45], [46]. Thus, one interpretation of our findings is that the strength of interference decays more rapidly with distance along chromosomes in TRIP13-defective cells as a consequence of altered properties of axes and/or other higher order chromosome structures.

Strikingly, the distribution of MLH1 foci along chromosomes is altered in *Trip13^mod/mod^* mutant spermatocytes, with the general effect that positional biases seen in wild type were less pronounced in the mutant. This is an unusual phenotype—to our knowledge, the only other mouse mutations reported to cause large-scale alterations in focus positions are hypomorphic alleles of *Mre11* or *Nbs1* [47]. The tendency of larger chromosomes to have interstitial regions “filled in” when there were two MLH1 foci on a bivalent could reflect the decreased average interference distance discussed above. However, interfocus distances are not relevant when considering chromosomes with single foci, and such chromosomes also showed a tendency toward more even distributions. We thus infer that the altered MLH1 focus distributions are not simply a consequence of altered interference. Instead, we speculate that the shortened SCs, altered interference distances, and altered CO distributions are all separate outcomes of a common underlying defect, i.e., altered axis structure. Recent studies of chromosome structure mutants in *C. elegans* provide precedent for such linked defects [45], [46], [48].

### TRIP13 is required for normal development of higher-order chromosome structures

TRIP13 is needed for formation of complete, structurally normal SC, as indicated by three defects in *Trip13* mutants. First, and most strikingly, three quarters of the autosomal bivalents from *Trip13^sev/sev^* mutant spermatocytes and oocytes showed substantial synaptic anomalies, especially pericentric forks. In human spermatocytes, pericentric regions often show delayed synapsis, indicating that SC elongation across these regions is slow or inefficient [49]. TRIP13 may be especially important for SC formation in such “difficult” regions. Possible molecular functions for TRIP13 in this process could include promoting efficient assembly of SC components, remodeling chromosome axes to a form permissive for SC elongation, or indirectly promoting SC formation via effects on recombination.

A second defect is that SCs were shorter on average in *Trip13^mod/mod^* spermatocytes than in wild type. The *Trip13^mod^* allele thus joins a growing list of mouse mutations that alter the lengths of SC and/or chromosome axes, including *Smc1β^–/–^* (shorter SCs) [50], and *Sycp3^–/–^* and *Spo11^+/–^Atm^–/–^* (longer SCs) [23], [51]. The cause(s) of altered lengths in these mutants is not known, but could involve changes in number and length of chromatin loops that emanate from chromosome axes, or changes in the longitudinal compaction of bases of chromatin loops [52].

Third, TRIP13 is needed for depletion of HORMADs from chromosome axes soon after synapsis [20] (Figure 5). Effects of TRIP13 on the structure of chromosome axes is a possible unifying theme for the SC defects in the mutants, which, as discussed above, may also connect other aspects of TRIP13 function.

### Conservation of TRIP13/Pch2 roles in recombination and chromosome structure

Thus far, budding yeast and mouse are the only organisms in which Pch2/TRIP13 is known to be required during normal meiosis, and there are a number of similarities—and some interesting differences—between the meiotic phenotypes of mutants in the two species. (Although currently available data do not indicate roles for PCH2 orthologs in unperturbed meiosis in *C. elegans* or *D. melanogaster*, it is possible that such roles may emerge upon further study.) Importantly, Pch2/TRIP13 is required in both yeast and mouse for normal meiotic recombination. In yeast, CO interference is weakened and closely spaced double crossovers occur more frequently than normal [14], [15], and in mouse, we find that inter-CO distances tend to be shorter. Both organisms show defects in forming an obligate CO/chiasma [15]. Additionally, *pch2* mutant yeast show defects at multiple steps in recombination, including early steps at or prior to DSB resection, although possible roles of Pch2 in efficient assembly of Rad51 or Dmc1 complexes have not been examined in yeast [9], [12]–[15].

Comparisons between the two species are more complicated when considering effects of Pch2/TRIP13 deficiency on CO numbers. By tetrad analysis, some genetic intervals in yeast show increased crossing over in *pch2* mutants [14], [15]. On its face, this pattern appears different from that in mouse, where we observed globally reduced CO numbers (MLH1 foci). However, the following points are noteworthy. First, not all intervals in yeast had increased crossing over, as some were unchanged in a *pch2* mutant [14]. Second, *pch2* mutation caused decreased CO numbers in some (but not all) intervals in a strain background that experiences fewer DSBs globally because of a hypomorphic *spo11* mutation [15]. Third, CO formation was reduced and delayed when assayed by direct physical analysis of DNA at the *HIS4LEU2* recombination hotspot [9]. Finally, our analysis of MLH1 focus distributions suggests that many genomic regions in mouse experience an *increase* in CO frequency in *Trip13* mutants, even though global numbers are decreased (Figure 10). Thus, although it is not yet clear whether *pch2/Trip13* mutations cause entirely congruent defects in crossing over in yeast and mouse, both organisms nonetheless are seen to have regionally variable alterations in CO number and placement.

Pch2/TRIP13 is also required in both organisms for normal dynamics of higher order chromosome structures, especially depletion of Hop1/HORMADs from all (mouse) or a subset (yeast) of axes where synapsis has occurred [9], [20]. Both organisms also show altered SC length, albeit with opposite directions for the net change (SCs are longer in *pch2* mutant yeast [14]).

The available data thus strongly support the conclusion that the mammalian and yeast proteins have roles in multiple aspects of both meiotic recombination and the development of higher order chromosome structures that are themselves closely integrated with recombination. These findings suggest that many specific roles of Pch2/TRIP13 are more widely conserved than previously appreciated.

## Materials and Methods

### Ethics statement

Experiments conformed to relevant regulatory standards and were approved by the MSKCC Institutional Animal Care and Use Committee.

### Mutant mice

Mouse embryonic stem cell lines RRB047 (Baygenomics, USA) and CH0621 (Sanger Institute, UK) (both cell lines from strain 129/Ola) were used to generate the *Trip13* mutant strains in this study. These cell lines contain a gene trap located in the second (CH0621) or third (RRB047) intron of the *Trip13* gene, both of which create an in-frame fusion of TRIP13 and a β-geo reporter. The exact locations of the gene traps were determined by PCR and subsequent sequencing of the *Trip13* locus. The ES cell lines were injected into blastocysts and transferred to pseudo-pregnant mice. Colonies derived from the resulting chimeric mice were maintained with a C57Bl/6×129/Sv mixed background. To minimize variability from strain background, experimental animals were compared to controls from the same litter or from the same matings involving closely related parents. Genotyping was performed by PCR of tail tip DNA as previously described [19], with the following conditions: 2 min at 94°C, then 40 cycles of 20 s at 94°C, 30 s at 59°C, and 35 s at 72°C. The *Trip13^mod^* allele was detected with primers TRIP13 GeNo F4 (5′-CGGGCCCTATCTTTTCAGTTCC), TRIP13 GeNo R5 (5′-CTATAGTGCCCTTAGGCTCAGG), and RRB047 End F2 (5′-TAACCCACTCGTGCACCCAACT), which amplify fragments of 1115 bp for the wild-type allele and 1253 bp for the mutant. The *Trip13^sev^* allele was scored using primers SIGTR621 F2 (5′-CTCAGAGAAGACCCAAAGCACAGT), SIGTR621 R2 (5′-CCAGGGCACATCAGTAGGAAGC), and RRB047 End F4 (5′-GCGGTTAGCTCCTTCGGTCCTC), yielding amplification products of 319 bp for wild type and 642 bp for mutant.

### RNA and protein expression analysis

RNA from adult and juvenile mouse testes was obtained using an RNeasy Mini kit (Qiagen) and cDNA was produced using SuperScript III First-Strand Synthesis System (Invitrogen) using oligo dT as primers. *Trip13* transcripts were amplified using the primers TRIP13 2A (5′-TGCAGCGCAGCGGAAGCACTGC) and TRIP13 1B (5′-CCACTGAGGCCCTCACTCTTCCTT), which amplify most of the full-length transcript. β-actin was amplified as a control using primers β-actin1 (5′-AGGTCTTTACGGATGTCAACG) and β-actin2 (5′-ATCTACGAGGGCTATGCTCTC). RNA in situ hybridization was performed on testis sections as described [53], using a 416-bp fragment of the *Trip13* transcript amplified using primers TRIP13 1A (5′-GTTTGTTCTGATTGATGAGGTGGA) and TRIP13 1B and labeled with [α^32^P]-UTP.

To separate different cell types using FACS, testes from adult mice were processed as described elsewhere [54]. Briefly, cells were liberated from testes by enzymatic treatment with collagenase, trypsin and DNase, then the resulting cell suspension was stained with Hoechst 33342 (Sigma) and cells were sorted using a MoFlo cytometer (Dako) with a 350 nm argon laser. Different cell types were separated based on DNA content and chromatin complexity [54]. Cellular composition was confirmed by spreading and immunofluorescence (data not shown).

### Cytology and histology

For cytology, testes from 2–4 month-old mice were prepared for surface spreading and sectioning as previously described [23], [55]. Prenatal ovaries were collected from 18.5–20.5 days post-coitum embryos and processed to obtain oocyte spreads as described [55]. Ovaries from 21-day-old mice were also recovered for histological analysis as described [19]. Immunofluorescence was performed using previously described methods [56] and antibodies [20], [23]. Additional primary antibodies used were goat anti-ATR (Santa Cruz), 1∶130 dilution; rabbit anti-BRCA1 (generously provided by C. Deng, NIH) [57], 1∶200 dilution; rabbit anti-RPA (generously provided by P. Cohen, Cornell University), 1∶100 dilution; and a rabbit anti-DMC1 (Santa Cruz, H-100), 1∶300 dilution. For RAD51, DMC1, RPA, and MLH1 focus counts, nuclei were staged using the extent of SYCP3 staining and synapsis as markers for meiotic prophase progression. Only foci that co-localized with the chromosome axes were counted. SC length and distances between the MLH1 foci on the SCs were evaluated as previously described [23].

Statistical tests were as described in the text. Mann-Whitney tests were applied to MLH1 focus numbers to avoid assuming normal distribution, but similar conclusions about statistical significance were obtained if Student t-tests were performed. To evaluate differences between wild type and mutants for MLH1 focus distributions along chromosomes without prior assumptions about the shape of those distributions, we used the Anderson-Darling k-sample test (from the “adk” package in R, version 2.9.2) [58], [59]. (Note that the goal was not to compare means of these distributions, but to compare their overall shape). Similar conclusions about significance of the difference between wild type and *Trip13^mod/mod^* homozygotes, and about the lack of significance of difference between wild type and *Trip13^+/mod^* heterozygotes, were obtained if the Kolmogorov-Smirnov test was used instead (data not shown).

For histological analysis, testis and ovarian sections were stained with periodic acid-Schiff (PAS) using hematoxylin as a counterstain. TUNEL staining was performed as previously described [60]. Staging of the PAS-stained testis sections was performed as described elsewhere [61].

## Supporting Information

Figure S1 Altered MLH1 inter-focal distances in *Trip13^mod/mod^* spermatocytes. Distances between pairs of adjacent MLH1 on autosomes of pachytene spermatocytes are plotted as in Figure 9, but with distances measured in mm of SC. Panels A–E show the frequency distributions (step plots) of inter-focus distances for wild type (blue) and *Trip13^mod/mod^* (red). Panels F–J show cumulative plots of the same data, with the number of chromosomes analyzed and the Kolmogorov-Smirnov test p value indicated. The left column of graphs (panels A, F) pools data for all autosomes. The remaining columns show data for groups of similarly sized chromosomes, ranked from largest to smallest. Autosome size ranks 17–19 are excluded from this analysis because they rarely have more than a single MLH1 focus.
(0.21 MB TIF)

Figure S2 Comparison of autosomal CO distributions in wild-type, *Trip13^+/mod^*, and *Trip13^mod/mod^* spermatocytes. Positions of MLH1 foci on spread chromosomes from pachytene spermatocytes are plotted as in Figure 10, but with data for wild type (dark blue) and *Trip13^+/mod^* (light blue) plotted separately for comparison. No statistically significant differences were observed between these genotypes for any of the comparisons described in the main text. Moreover, all conclusions about statistical significance were the same whether *Trip13^mod/mod^* was compared with wild type only or with the pooled wild type and heterozygote data.
(0.80 MB TIF)
