# Supplementary figures and images for: Mouse TRIP13/PCH2 Is Required for Recombination and Normal Higher-Order Chromosome Structure during Meiosis
Source: PLoS Genet. 2010 Aug 12;6(8):e1001062. doi: 10.1371/journal.pgen.1001062 (PMC2920839; doi:10.1371/journal.pgen.1001062)

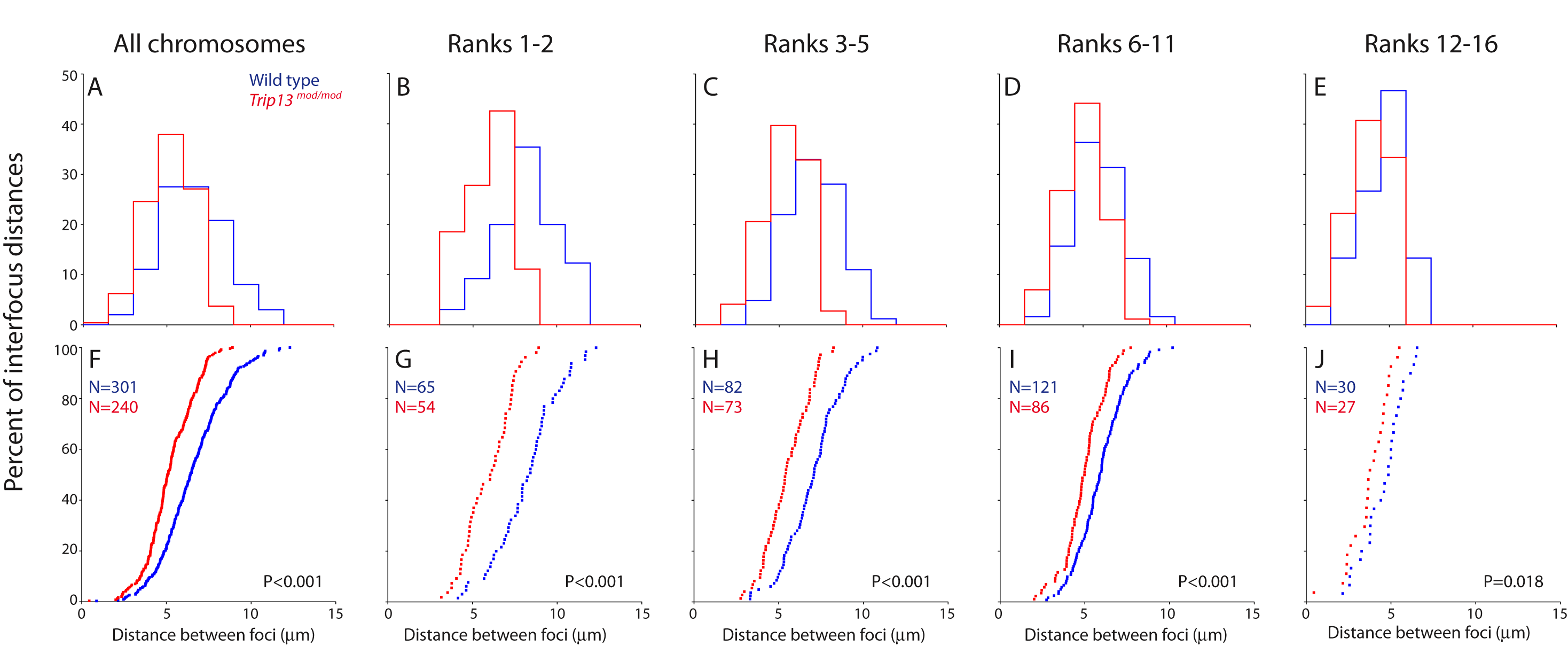

Supplement: Figure S1 — Altered MLH1 inter-focal distances in Trip13mod/mod spermatocytes. Distances between pairs of adjacent MLH1 on autosomes of pachytene spermatocytes are plotted as in Figure 9, but with distances measured in mm of SC. Panels A–E show the frequency distributions (step plots) of inter-focus distances for wild type (blue) and Trip13mod/mod (red). Panels F–J show cumulative plots of the same data, with the number of chromosomes analyzed and the Kolmogorov-Smirnov test p value indicated. The left column of graphs (panels A, F) pools data for all autosomes. The remaining columns show data for groups of similarly sized chromosomes, ranked from largest to smallest. Autosome size ranks 17–19 are excluded from this analysis because they rarely have more than a single MLH1 focus. (0.21 MB TIF) [file pgen.1001062.s001.tif]

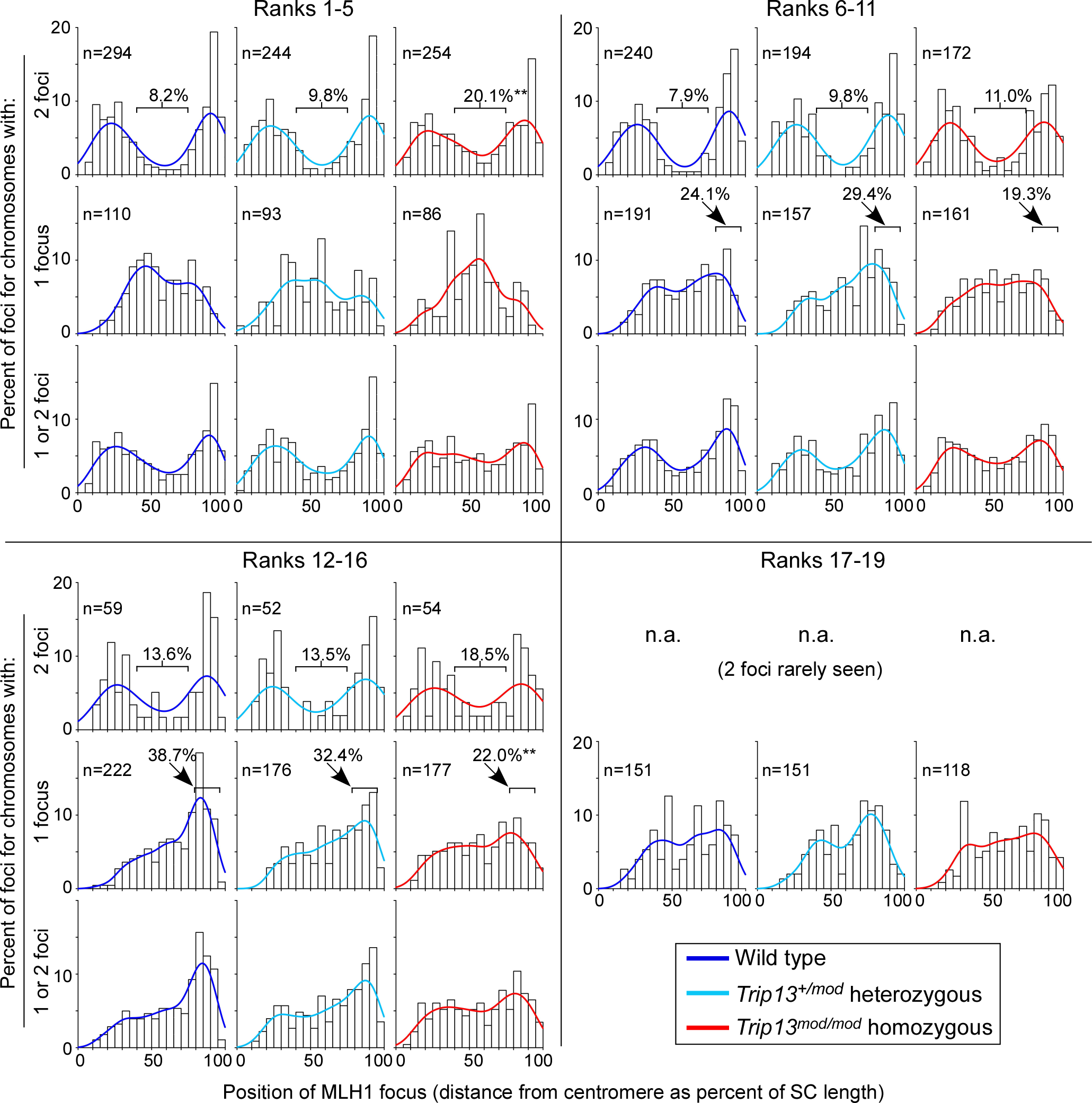

Supplement: Figure S2 — Comparison of autosomal CO distributions in wild-type, Trip13+/mod, and Trip13mod/mod spermatocytes. Positions of MLH1 foci on spread chromosomes from pachytene spermatocytes are plotted as in Figure 10, but with data for wild type (dark blue) and Trip13+/mod (light blue) plotted separately for comparison. No statistically significant differences were observed between these genotypes for any of the comparisons described in the main text. Moreover, all conclusions about statistical significance were the same whether Trip13mod/mod was compared with wild type only or with the pooled wild type and heterozygote data. (0.80 MB TIF) [file pgen.1001062.s002.tif]
